# Polyacrylamide and Polyacrylamide/Polysaccharide Hydrogels for Well Water Shutoff in High-Temperature Reservoirs

**DOI:** 10.3390/gels11110862

**Published:** 2025-10-28

**Authors:** Aleksey Telin, Natalia Sergeeva, Rustem Asadullin, Ekaterina Gusarova, Ravil Yakubov, Vladimir Dokichev, Anatoly Politov, Elina Sunagatova, Natalia Gibadullina, Galina Teptereva, Lyubov Lenchenkova

**Affiliations:** 1 Ufa Scientific and Technical Center, LLC, 99/3, Kirova Street, 450078 Ufa, Russia; 2 Faculty of Mining and Petroleum, Ufa State Petroleum Technological University, 1, Kosmonavtov Street, 450064 Ufa, Russia; yorkerbridge@mail.ru (R.A.); teptereva.tga@yandex.ru (G.T.);; 3 Interdisciplinary Research Laboratory of Oilfield Chemistry, Ufa University of Science and Technology, 12, Karla Marksa Street, 450008 Ufa, Russia; 4 Ufa Institute of Chemistry, Ufa Federal Research Center, Russian Academy of Sciences, 71, Oktyabrya Avenue, 450054 Ufa, Russia; 5 Institute of Solid State Chemistry and Mechanochemistry of Siberian Branch RAS, 18, Kutateladze Street, 630128 Novosibirsk, Russia

**Keywords:** hydrogels, water shutoff, high-temperature reservoirs, polyacrylamide, polysaccharides, paraform crosslinking, rheological properties

## Abstract

Polyacrylamide and polyacrylamide/polysaccharide hydrogels exhibiting high structural and mechanical properties, along with acceptable gelation times and gelant viscosity, are proposed for water shutoff applications in high-temperature reservoirs. The obtained polyacrylamide gels demonstrate an elastic modulus 1.6–2.7 times higher than that of the baseline polyacrylamide–resorcinol–paraform–sulfamic acid gel (17.2 Pa), reaching up to 46.3 Pa, while the polyacrylamide/polysaccharide gels surpass it by a factor of 2.3–5.2, reaching up to 89.9 Pa. The gelation time of the polyacrylamide/polysaccharide gels ranges from 3 to 7 h, with the gelant viscosity varying from 685 to 2098 mPa·s at a shear rate of 100 s^−1^. Crosslinking of polyacrylamide with polysaccharides was achieved using paraform. Using the gel based on crosslinked polyacrylamide with xanthan as an example, spectral methods characterized the copolymer constituting the basis of the plugging material. Our analysis established that crosslinking occurs between the amide group of polyacrylamide and the hydroxyl group of the polysaccharide. Model reactions with low-molecular-weight analogs (glucose, acetamide, and formaldehyde), coupled with mass spectrometric confirmation of the structure of the resulting products, revealed possible reaction pathways. The crosslinking of polyacrylamide was investigated using a broad range of polysaccharides of plant and microbiological origin. The resulting series of hydrogels, possessing the suite of properties required for water shutoff in high-temperature formations, will enable oil companies (operators) and service firms to select a specific gel-forming system based on project objectives, logistics, and budget constraints.

## 1. Introduction

Well water shutoff using hydrogels in high-temperature formations has become one of the promising and rapidly developing methods for extending the cost-effective production life of wells over the last 10 years [1,2]. Organic crosslinking agents are preferred, as they provide higher strength and thermal stability of hydrogels [3,4]. For instance, polyacrylamide and hexamethylenetetramine gels used for water shutoff at high temperatures (85 to 120 °C) exhibit high strength properties and acceptable gelation times [5,6].

The addition of bentonite to a polyacrylamide gel crosslinked with an organic crosslinker facilitates the formation of a robust three-dimensional network structure, significantly enhancing the gel’s resistance to shear loads. Increasing the bentonite content also augments the gel’s viscoelastic properties [7].

An amphiphilic polymer based on acrylamide, sodium acrylate, and N-dodecylacrylamide, upon interaction with a molecular complex of phenol-β-cyclodextrin, undergoes crosslinking at a reduced rate under high-temperature conditions due to the gradual release of phenol from the β-cyclodextrin ring. The resulting gel possesses high thermal and salt resistance, as well as shear resistance [8]. Moreover, studies established that phenol release from the β-cyclodextrin cavity occurs through competitive interaction with the dodecyl radical at the polymer’s nitrogen atom.

A viscoelastic gel for well water shutoff in high-temperature formations (>100 °C) was obtained from a copolymer of acrylamide and tert-butyl acrylate crosslinked with polyethyleneimine; its elastic modulus is significantly higher than that of known hydrogels crosslinked with inorganic crosslinkers [4].

In study [9], the authors conducted experiments enabling the derivation of an exponential equation to predict the gelation time of a thermally stable copolymer of acrylamide and 2-acrylamido-2-methylpropanesulfonic acid crosslinked with hexamethylenetetramine in the presence of hydrochloric acid (as an initiator); they revealed that the initiator concentration has a decisive influence on the gelation time. Article [10] describes the preparation of weak gels based on polyacrylamide and resorcinol for enhanced oil recovery, featuring ultra-long gelation times (up to 47 days) at 45 °C. Based on the van ’t Hoff rules, it can be assumed that at 90 °C, the gelation time would decrease by approximately 10 times, becoming quite acceptable for application in high-temperature, low-permeability formations.

The authors of work [11] demonstrated that hydrogels based on sulfonated polyacrylamide and crosslinkers of organic (polyethylene hexamethylenetetramine) and inorganic [Cr(CH_3_COO)_3_ and Al(NO_3_)_3_∙9H_2_O] nature maintain stability up to 100 °C, with organically crosslinked gels exhibiting greater thermal stability than those crosslinked with inorganic agents. Review articles [12,13,14] also emphasize that gels crosslinked with organic agents hold a significant advantage in strength and thermal stability over those crosslinked with inorganic agents. Furthermore, review [14] notes that the use of polyethyleneimine as a crosslinker also ensures the environmental friendliness of the water shutoff process.

Article [15] established that a polymer-gel system based on polyacrylamide crosslinked with potassium dichromate operates successfully at temperatures above 85 °C; however, the high toxicity of potassium dichromate makes this gel less attractive for well water shutoff.

Work [16] showed that additives of alkanolamines and quaternary ammonium salts to polyacrylamide gels crosslinked with polyethyleneimine allow for reducing the concentration of the expensive crosslinker while maintaining the strength properties of the plugging material. Over 100 well treatments in the Gulf of Mexico shelf have been performed using a copolymer of acrylamide with tert-butyl acrylate crosslinked with polyethyleneimine; notably, in fractured carbonate formations, the gel was reinforced with a cement-like material at the end of injection. The temperature range of the water shutoff targets was from 27 to 127 °C; the authors emphasize that most treatments were conducted with high cost-effectiveness.

Besides polyacrylamide gels, polysaccharide gels are also successfully used for well water shutoff. For example, works [13,17,18] demonstrate the effective application of modified starch in fractured and granular reservoirs. It is important to note that polysaccharide gels exhibit high resistance to salt aggression [19,20]. Article [21] describes the synthesis of a novel hydrogel with semi-interpenetrating networks based on partially hydrolyzed polyacrylamide and scleroglucan, crosslinked with chromium acetate and resistant to formation brine salinity and temperature.

Copolymers of polysaccharides with polyacrylamide are most commonly obtained by grafting polyacrylamide blocks onto the polysaccharide. This is achieved through radical polymerization of acrylamide monomer in the presence of an initiator and polysaccharide, with N,N′-methylenebisacrylamide used as the crosslinker [22].

It should be noted that the theory and practice of polysaccharide-grafted copolymers are well-studied [23]. Various polysaccharides are used for water shutoff via acrylamide-grafted copolymers, such as welan gum [24], chitosan [25], and starch [20]. Exceptionally strong gels based on calcium alginate with grafted polyacrylamide, crosslinked with N,N′-methylenebisacrylamide, are formed due to dual crosslinking [26]. Hydrogels with excellent mechanical properties are obtained from κ-carrageenan and polyacrylamide through dual crosslinking with zirconium ions; here, dual crosslinking occurs via coordination bonds between κ-carrageenan/polyacrylamide and the metal ion [27]. Such superior structural and mechanical properties of polyacrylamide/polysaccharide copolymer-based gels are explained by the combination of rigid heterochain polysaccharides with flexible fragments of the carbon-chain polyacrylamide, crosslinked into a single macromolecule.

Thus, based on a generalization of the literature data concerning the application of hydrogels for water shutoff in high-temperature reservoirs, the following conclusions can be drawn. Effective plugging compositions based on sulfonated thermally stable polyacrylamides and mixed polyacrylamide/polysaccharide compositions have been developed and tested. For sulfonated polyacrylamide systems, the key disadvantage is the high cost of the polymer, while for polyacrylamide/polysaccharide gels, the drawback lies in the complexity of the graft copolymer synthesis.

Based on the classic monograph by J. Frederic Walker on the chemistry and technology of formaldehyde [28] (1953), it can be assumed that formaldehyde (paraform) would crosslink polysaccharides with polyacrylamide via methylol derivatives. Unfortunately, we were unable to find the literature on this specific method of synthesizing polysaccharide-polyacrylamide copolymers; however, publications on crosslinking polysaccharides with proteins using formaldehyde are known [29].

The aim of the present work is to develop polyacrylamide and polyacrylamide/polysaccharide hydrogels for water shutoff operations in high-temperature formations, featuring a comfortable gelation time (>3 h) and high structural–mechanical characteristics alongside the development of a simple, field-friendly implementation method utilizing inexpensive and readily available components.

## 2. Results and Discussion

Over the past several years, our research group has conducted water shutoff operations using a reliable and technologically advanced hydrogel system based on polyacrylamide–resorcinol–paraform in both granular [30] and fractured [31] reservoirs at moderate temperatures (up to 50 °C). However, when the temperature increases to 60 °C and above, this polymer–crosslinker system begins to crosslink rapidly, making it impossible to deliver the gelant to the target isolation interval. We patented a composition offering a comfortable gelation time by introducing a crosslinking retarder, sulfamic acid, into the established system [32]. Unfortunately, this modification resulted in degraded structural and mechanical properties of the resulting plugging material. Specifically, a gel containing 1.7% polyacrylamide, 0.15% paraform, and 0.05% resorcinol exhibited an elastic modulus of 22.6 Pa; whereas a gel of the same composition with the addition of 0.2% sulfamic acid showed a reduced elastic modulus of 17.2 Pa (Table 1, Experiment 1). We adopted the structural and mechanical properties of this practically applied hydrogel system as the baseline for comparison.

When resorcinol in the baseline gel was replaced with hydroquinone or pyrocatechol, while maintaining the same concentrations of the other components, the elastic modulus increased to 35.4 Pa and 31.5 Pa, respectively (Experiments 2 and 3). Notably, for the composition containing hydroquinone, the addition of 0.1% Na_2_CO_3_ was required to achieve gelation. The gelation time for the hydroquinone-based composition was 6.5 h, and 7 h for the pyrocatechol-based composition.

We hypothesized that lignosulfonates, possessing bulky molecules capable of condensing with paraform [33,34], could provide acceptable gelation times at 90 °C and satisfactory structural and mechanical properties when used instead of resorcinol. Indeed, a system comprising polyacrylamide (1.7%), paraform (0.3%), lignosulfonate (0.1%), and Na_2_CO_3_ (0.1%) crosslinked within 4.5 h and achieved an elastic modulus of 27.9 Pa (Experiment 4).

Increasing the paraform content to 0.5% and the lignosulfonate content to 0.2%, while keeping the polyacrylamide and Na_2_CO_3_ concentrations unchanged, yielded a gel with an elastic modulus of 30.1 Pa (Experiment 5) and a gelation time of 4 h.

Subsequently, we substituted lignosulfonate with condensed sulfite spent liquor (CSSL), neutralized with sodium hydroxide (added at 0.2% in the plugging composition). This resulted in a gel with an elastic modulus of 33.6 Pa (Experiment 6) and a gelation time of 3 h. (For reference: Sulfite-alcohol spent liquor is a by-product of the forest chemical industry, generated during the sulfite pulping of wood cellulose; CSSL is the product of condensing sulfite-alcohol spent liquor with formaldehyde).

Finally, we modified the plugging composition to include only one crosslinker—paraform. By increasing its concentration to 0.4% and the polyacrylamide concentration to 1.9%, we obtained a corresponding gel after 7 h at 90 °C with an elastic modulus of 46.3 Pa (Table 1, Experiment 7).

As evident from Table 1, the polyacrylamide gels developed for application in high-temperature formations possess good structural and mechanical properties that surpass the baseline gel, coupled with a comfortable gelation time. These gels show promise for successful use in Neocomian and Jurassic deposits of Western Siberian fields, where formation temperatures range from 80 to 95 °C [35].

**Figure 1:**
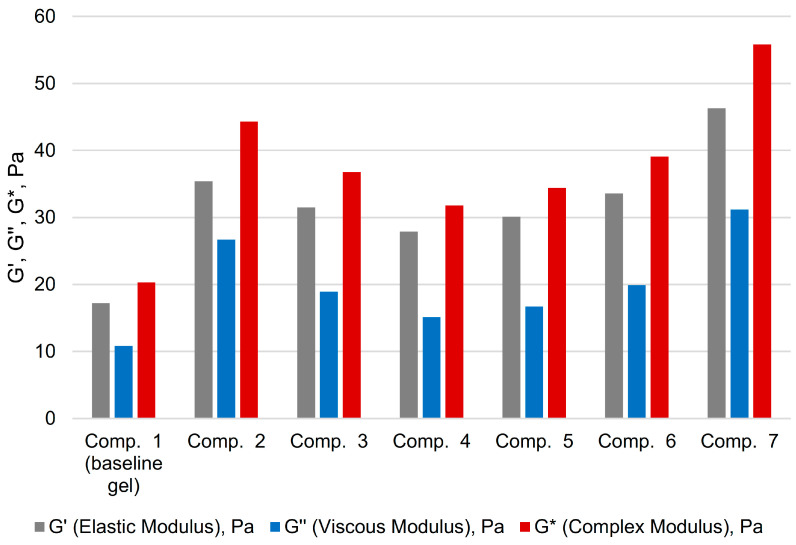
Bar chart of polyacrylamide gels oscillatory test results.

For clarity and to facilitate the perception of the numerical data, a bar chart is provided (Figure 1).

Given that forestry chemicals are classified as low-hazard substances [36], gels crosslinked with lignosulfonates and CSSL contain only one toxic component—paraform. The same applies to the polyacrylamide gel crosslinked solely with paraform. Moreover, the rheological properties of the gel crosslinked with paraform alone surpass those obtained with two crosslinkers. Specifically, this gel exhibited an elastic modulus of 46.3 Pa, a viscous modulus of 31.2 Pa, and a complex modulus of 55.8 Pa. This circumstance is presumably related to the increased polyacrylamide concentration of 1.9%. In all other cases (Table 1), polyacrylamide was used at a concentration of 1.7%; however, at this reduced concentration (1.7%), no gel formation occurred when using only paraform as the crosslinker.

We additionally noted an interesting observation: not all gel-forming systems successfully crosslinked with the sample of polyacrylamide grade A523; whereas complete gelation occurred with the sample of polyacrylamide grade EOR-1141 when using lignosulfonate and CSSL. Based on our experience with the polyacrylamide–resorcinol–paraform gel-forming system, it was established that these polymer grades are interchangeable and possess similar molecular weight and degree of hydrolysis values. Specifically, sample A523 has a molecular weight of 7.2 million Da and a degree of hydrolysis of 22%, while sample EOR-1141 has a molecular weight of 7.8 million Da and a degree of hydrolysis of 24%. Although the difference seems minor, it proved significant for the crosslinking of these polyacrylamide samples with forestry chemicals and paraform.

It should be noted that a comfortable gelation time of three hours was selected based on our operational experience in long-reach horizontal wellbores utilizing hydraulic dual-packer assemblies.

The gelation process (Sydansk gel test [37]) for the composition PAM 1.9% (4 g), PFA 0.4% (0.84 g), Water 97.7% (205.68 g) is visualized in Figure S1.

Regarding the behavior of hydrogels under mechanical stress, it is important to highlight that this area is well-studied [38,39], particularly in biomedical applications. The most frequently conducted tests are tensile, compression, indentation, and torsion tests.

We performed both qualitative and quantitative mechanical tests on the obtained hydrogels. Qualitative mechanical testing was conducted on polyacrylamide crosslinked with paraform. The gel was placed in a custom device and stretched from 3 cm to 30 cm (Figure S2). A total of five stretch cycles was performed. As can be seen, the filament returns to its original length after release and can be stretched again to 30 cm, i.e., a tenfold elongation, indicating the high elasticity of the gel. Figure S2 shows that five cycles of stretching and compression did not affect the visible elastic properties of the polymer filament. The theory of stretching and rupture of viscoelastic polymer filaments is described in work [40].

Quantitative assessment of the hydrogel sample’s stability under significant mechanical stress and evaluation of its rheological parameters post-treatment was conducted directly in the rheoviscometer measuring cell. For this purpose, an aliquot was taken, and the rheological parameters of the gel sample were determined six times consecutively without extracting it from the measuring system (cell). The hydrogel in the cell was subjected to mechanical stress due to the oscillation amplitude of the measuring system. The absolute value of the maximum rotation angle in the first measurement was 327°. In subsequent measurements, it did not drop below 130°. Thus, it can be stated that the sample was subjected to significant mechanical stress throughout the entire measurement series, since the linear viscoelastic range (LVR) corresponded to a deflection angle not exceeding 3°, and the crossover point corresponded to a deflection angle of 4°.

The results are presented in Table 2.

**Figure 2:**
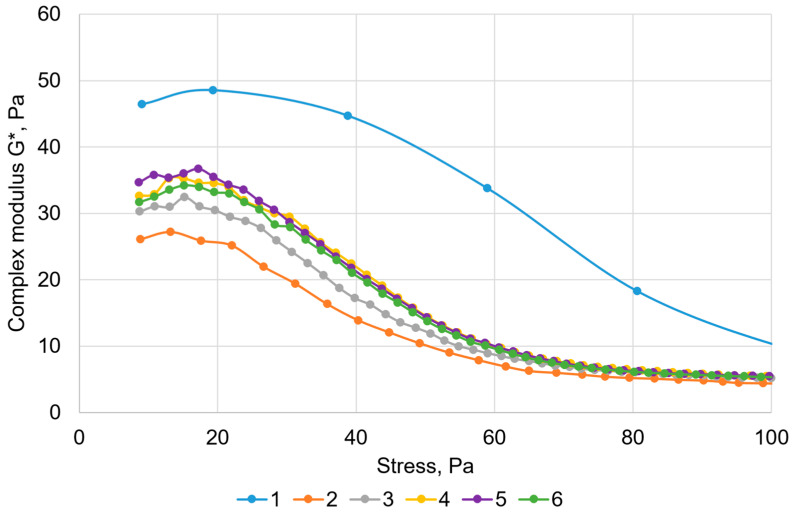
Oscillation test results for six repeated measurements.

Figure 2 shows the dependencies of the complex modulus on the shear stress for all 6 measurements.

As evident from Table 2, after the initial structural breakdown of the gel, its parameters stabilized in subsequent measurements and changed on average as follows: the complex modulus decreased by 35% (from 48.5 to 31.6 Pa), the crossover point value decreased by 56% (from 72.6 to 31.8 Pa), and the linear viscoelastic range decreased by 38% (from 38.8 to 23.9 Pa). It can be concluded that after mechanical treatment, the structural-mechanical properties of the gel decrease; however, complete breakdown does not occur, and it retains a significant portion of its viscoelastic properties. This phenomenon is explained by the fact that during the second determination, the loss of hydrogel strength occurs due to imperfections in the network, whose weak links break down under sample loading [41,42].

Upon closer examination, it can be noted that subsequent measurements (2 through 6) showed a slight increase in all rheological parameters. With further measurements, a slight recovery of the hydrogel is observed. This self-healing of the network structure is associated with the reformation of the network after the significant restructuring following the first determination, whereby a secondary network structure is formed via hydrogen bonds. Such behavior of polysaccharide gels has been described in work [43].

**Figure 3:**
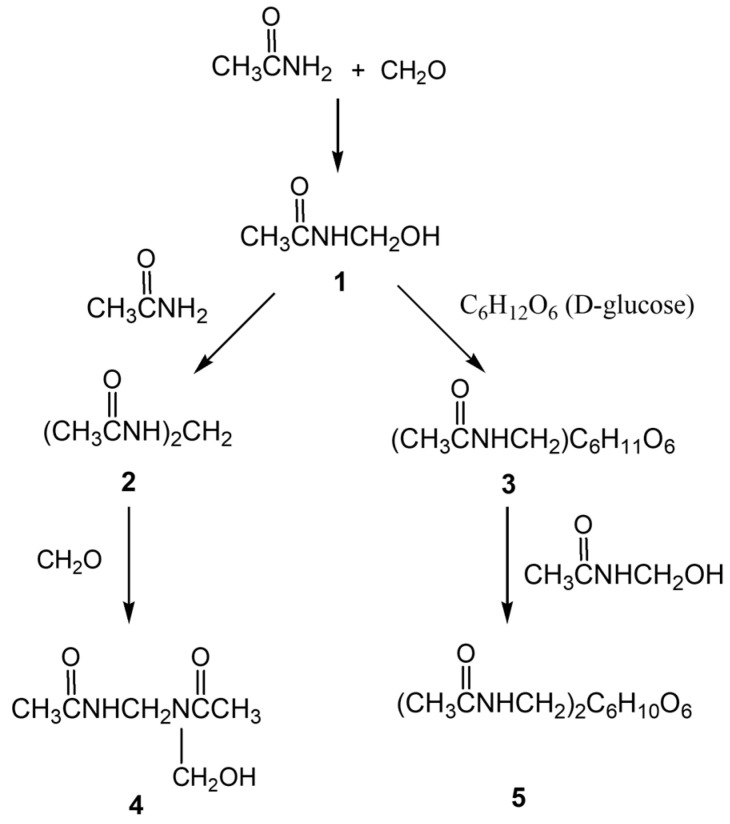
Reaction scheme of acetamide with D-glucose and formaldehyde.

At the outset of our research, we found no data in the scientific literature on the formation of hydrogels resulting from the crosslinking of polyacrylamide with polysaccharides using paraform. Therefore, to investigate the possibility of forming chemical bonds (crosslinks) between polyacrylamide and polysaccharide macromolecules via a methylene bridge, we conducted a model experiment with their low-molecular-weight analogs. Specifically, we studied the interaction of glucose with acetamide and formaldehyde, taken in a 1:1:1 molar ratio at 90 °C (Figure 3). As a result, it was established that the reaction of glucose with acetamide and formaldehyde proceeds with the formation of N-(hydroxymethyl)acetamide (1), N,N′-methylenediacetamide (2), (N-acetamidemethyl)-D-glucopyranose (3), N-(hydroxymethyl)-N,N′-methylenediacetamide (4), and di(N-acetamidemethyl)-D-glucopyranose (5) in a ratio of 15:20:11:1:6, respectively.

**Figure 4:**
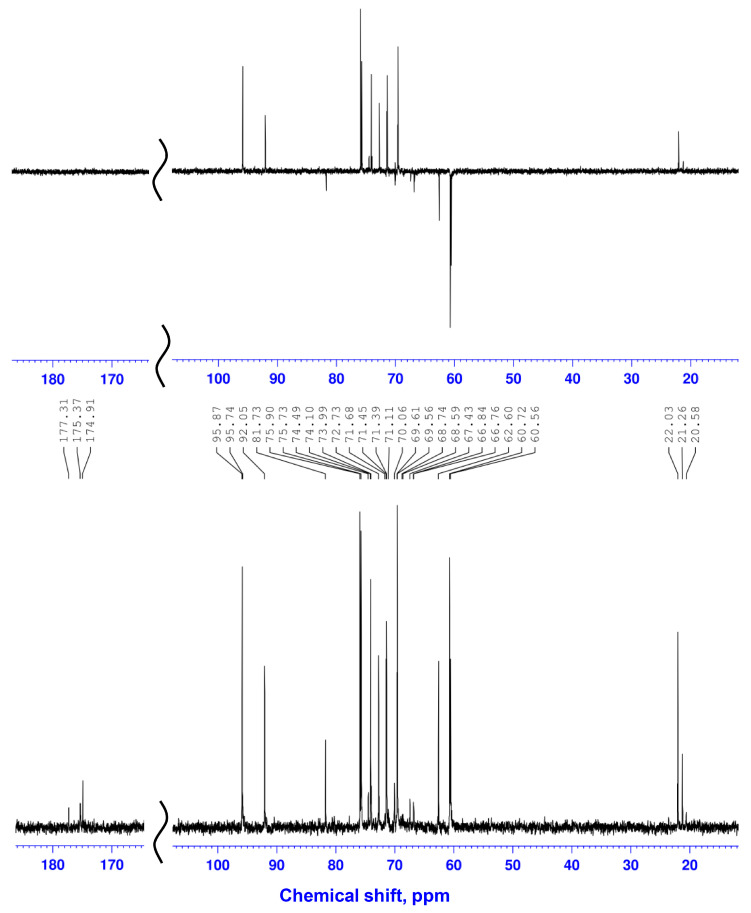
^13^C NMR spectrum of the reaction products of acetamide with D-glucose and formaldehyde.

The composition and structures of the resulting reaction products were studied using chromatography–mass spectrometry and NMR (Nuclear magnetic resonance) spectroscopy (Figures S3–S5 and Figure 4). Interpretation of the structures of the obtained compounds and assignment of the signals from H, C, and N atoms were performed using ^1^H, ^13^C, and ^15^N NMR spectroscopy, two-dimensional heteronuclear correlation {^1^H, ^15^N} HMBC spectra, and the known literature data [44,45,46,47].

Interpretation of the obtained mass spectrum, considering the reaction mechanism, allowed for the identification of the formed compounds (**1**–**5**) corresponding to the following (M + H)^+^ ions with *m*/*z* 90.0; 131.0; 143.0819; 252.1090 and 323.1452. Analysis of the mass spectrum of the crosslinking reaction products showed that for the compounds obtained, dehydration reactions are characteristic under APCI (Atmospheric Pressure Chemical Ionization) conditions. For example, the (M + H)^+^ ions 252.1090 and 323.1452, corresponding to compounds **3** and **5**, are accompanied in the mass spectrum by ions with *m*/*z* 234.0973 and 305.1, formed as a result of dehydration. The structures of the obtained compounds were confirmed by ^1^H, ^13^C, and {^1^H, ^15^N} HMBC NMR spectra (Figures S4 and S5 and Figure 4).

In the ^1^H NMR spectrum (Figure S4), characteristic signals of the CH_3_ groups of the acetamide fragments are observed as singlets at δ_H_ 1.879; 1.900; 1.923 and 1.931 ppm, belonging to six amide nitrogen atoms, which is unambiguously manifested in the {^1^H, ^15^N} HMBC NMR spectrum (Figure S5). Signals from the protons of the methylene groups NCH_2_ and OCH_2_ were recorded at δ_H_ 4.513; 4.529; 4.546; 4.554 and 4.563 ppm [44].

In the ^13^C NMR spectrum of the crosslinking reaction products of acetamide with D-glucose, signals from the carbonyl carbon atoms belonging to the amide fragments are observed at 177.31, 175.37 and 174.91 ppm, while the methyl groups appear in the region of 20.58–22.03 ppm. Signals at δ_c_ 62.60, 66.76, 66.84 and 67.84 ppm belong to the formed methylene groups at the amide nitrogen atoms (-CONHCH_2_-), which is unambiguously confirmed by the ^13^C{^1^H} DEPT 135 spectrum, where the signals of the methylene carbon atoms exhibit negative intensity (Figure 4). The wide range of signals belonging to the glucopyranose ring and hydroxymethyl groups at 66.76–75.91 ppm indicates that the crosslinking reaction proceeds via the hydroxyl groups of D-glucose [47].

Thus, the model reaction established that, alongside the crosslinking of acetamide molecules, crosslinking between the amide group of acetamide and the hydroxyl group of glucose also occurs. The obtained experimental data are of practical interest for the development of novel polyacrylamide gels with a three-dimensional network structure, formed as a result of chemical bond formation between polyacrylamide and polysaccharide molecules using paraform as a crosslinking agent.

Encouraged by the results of the model experiment, we proceeded to crosslink xanthan with polyacrylamide via paraform. The component composition of the reactants used for spectral analysis in D_2_O is given in Table 3.

**Figure 5:**
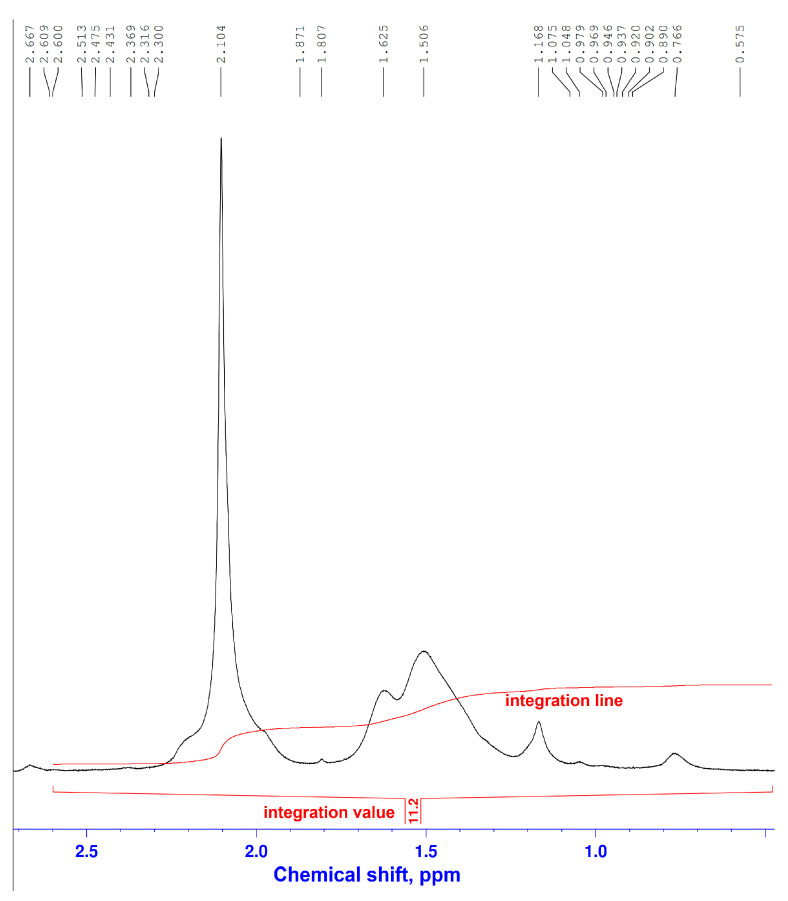
^1^H NMR spectrum of the gel.

**Figure 6:**
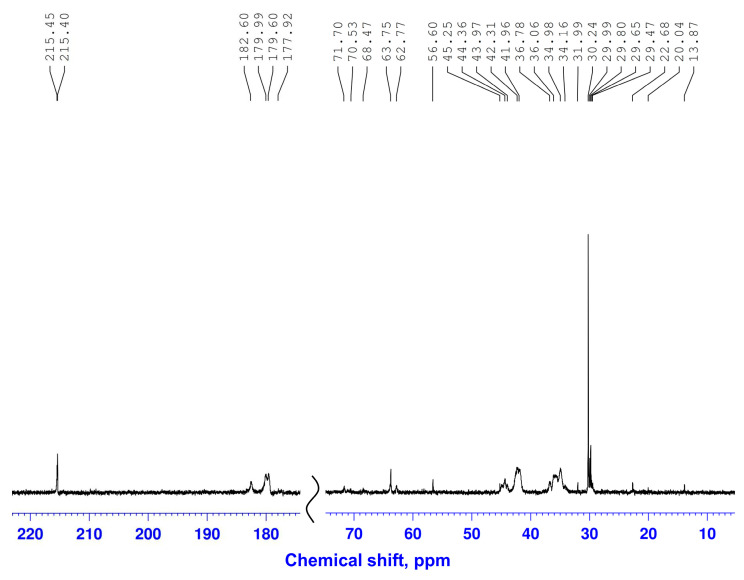
^13^C NMR spectrum of the gel.

The ^1^H and ^13^C NMR spectra of the obtained gel in D_2_O solution (Figure 5 and Figure 6, respectively) contain characteristic signals confirming the crosslinking of polyacrylamide with xanthan. The ^1^H NMR spectrum of the gel shows two groups of broadened signals at 1.506 and 2.104 ppm, belonging to the protons of the CH_2_ and CH groups of the polyacrylamide chain. Signals from the protons of the NCH_2_ and OCH_2_ methylene groups are recorded in the region of δ_H_ 3.22–3.713 ppm.

The ^13^C NMR spectrum of the gel contains sets of signals from CH_2_ and CH groups at 36–38 and 43.5–44.3 ppm, respectively, and broadened signals in the region of 177.92–182.60 ppm, belonging to the carbonyl carbon atoms of polyacrylamide and xanthan. Signals in the region of δ_c_ 62.77–63.75 ppm belong to the methylene fragments (-CONHCH_2_NHCO- and -CONHCH_2_O-), formed during the interaction of the amide nitrogen atoms of polyacrylamide with the hydroxyl groups of xanthan and paraform, which covalently link the macromolecules of polyacrylamide and polysaccharide [47].

**Figure 7:**
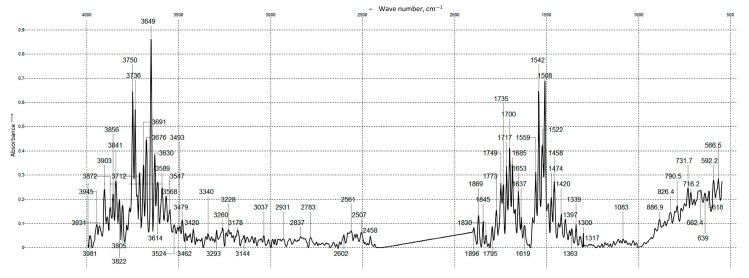
IR spectrum of the gel film.

The structure of the resulting polyacrylamide–xanthan–paraform hydrogel was further confirmed by IR spectroscopy data. The IR spectrum is characterized by intense bands of N-H and O-H stretching vibrations in the region of 3903–3479 cm^−1^ and intense absorption bands at 1869–1637 cm^−1^, corresponding to vibrations of carbonyl and ester groups (Figure 7).

The IR spectrum also shows intense absorption bands in the region of 1559–1508 cm^−1^, which, according to the literature data [48,49], are characteristic of substituted amides, and their formation may result from the intermolecular interaction of polyacrylamide with paraform and subsequent condensation of the amide group methylol groups with xanthan. Furthermore, peaks at 2931, 2837, 2783, 1458, 1420, and 1397 cm^−1^ represent absorption bands of asymmetric stretching, symmetric stretching, asymmetric bending, and symmetric bending vibrations of the C-H bonds in the methylene group [50].

The obtained FT-IR (Fourier-transform infrared spectroscopy) and NMR spectroscopy data indicate that the gel network structure is formed both through the crosslinking of xanthan with polyacrylamide via bridge formation (-CONHCH_2_O-) and as a result of inter- and intramolecular crosslinking of polyacrylamide macromolecules.

**Figure 8:**
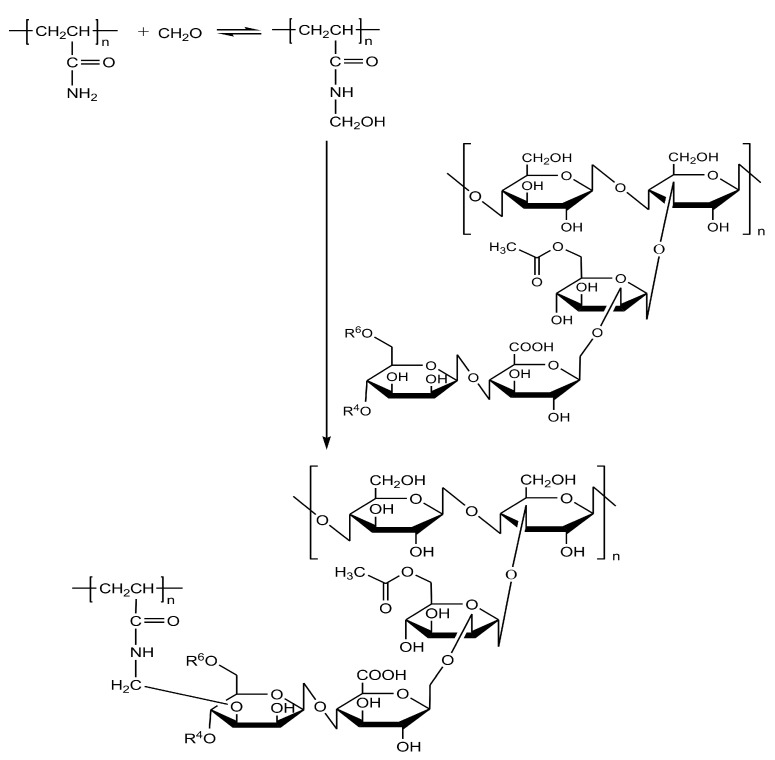
Crosslinking reaction of polyacrylamide and xanthan macromolecules (proposed mechanism).

Based on both the literature data and our experimental results, we propose the following mechanism for the crosslinking (formation of covalent chemical bonds) of polyacrylamide and polysaccharide macromolecules via a methylene bridge using paraform [44,46,47] (Figure 8). In the first stage of this two-step process, nucleophilic addition of the amide nitrogen atom of polyacrylamide to the carbonyl group of formaldehyde takes place, yielding poly(N-hydroxymethyl)acrylamide. Subsequent intermolecular interaction of the methylolamide fragment of poly(N-hydroxymethyl)acrylamide with the hydroxyl group of the polysaccharide, resulting in dehydration, leads to the formation of cross-linked poly(N-hydroxymethyl acrylamide)/polysaccharide.

The proposed mechanism of polysaccharide crosslinking with polyacrylamide via the formation of (-CONHCH_2_O-) bridges is supported by studies on the reactions of acetamide with formaldehyde [44], as well as acrylamide with formaldehyde and butanol [51]. The formation of an intermediate compound—N-hydroxymethylacetamide—in the interaction of formaldehyde with acetamide was experimentally confirmed and verified by NMR spectroscopy and mass spectrometry.

As can be seen from the reaction scheme (Figure 8), which is based on the interpretation of the obtained spectra, a hydrogel of the xanthan-polyacrylamide copolymer was formed. Having determined its rheological characteristics (Table 4, Experiments 1 and 2), we concluded that we had obtained a promising plugging material for well water shutoff in high-temperature formations. To assess the generality of the method for crosslinking polysaccharides with polyacrylamide via paraform, we tested a range of polysaccharides of plant and microbiological origin in this reaction (Table 4). As it turned out, all samples underwent crosslinking, although in some cases it was necessary to reduce the concentration of polysaccharides to ensure the gelant viscosity remained below 1000 mPa·s at 100 s^−1^. The latter requirement is related to the difficulty of injecting viscous gelants into the target isolation interval.

**Figure 9:**
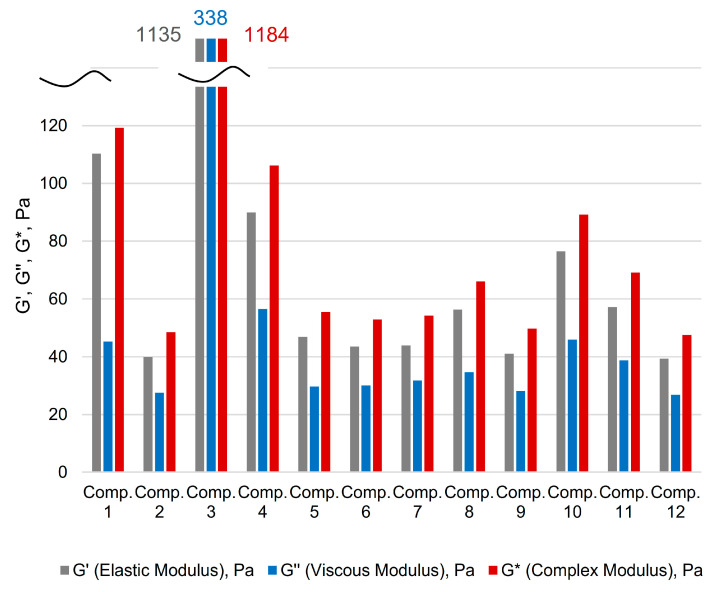
Bar chart of polyacrylamide/polysaccharide gels oscillatory test results.

For clarity and to facilitate the perception of the numerical data, a bar chart is provided (Figure 9).

Experiments on the crosslinking of polyacrylamide with xanthan and guar gum showed that at a concentration of both polymers of 1.9% and paraform of 1.6% (Table 4, Experiments 1 and 3), extremely strong gels are formed. However, the gelant viscosity reached 1128 and 2098 mPa·s, respectively. With such gelant viscosity, it is impossible to pump it into wells with horizontal completions and would be highly problematic for deviated wells. By reducing the polysaccharide concentration to 0.3% and paraform to 0.4%, we managed to obtain gels with the required characteristics (Table 4, Experiments 2 and 4).

When using CMC (carboxymethyl cellulose) (Table 4, Experiment 5) at a concentration of only 0.1%, the resulting gel was slightly inferior in rheological properties to gels using guar and xanthan but significantly surpassed pure polyacrylamide gels (Table 1). Two samples of carboxymethyl starch, widely used in well drilling (Table 4, Experiments 6 and 7), showed results close to CMC. In Experiments 8 and 9 (Table 4), mechanoactivated potato starch and corn starch were used as polysaccharides. In both cases, gels with the required properties were obtained, with the performance of the gel with potato starch noticeably surpassing that of the gel with corn starch. Using mechanoactivated corn flour and rice flour allowed increasing the concentration of the starch-containing material to 0.3%, as the gelant viscosity fit within the required range. At the same time, the elastic modulus increased significantly to 76.5 and 57.2 Pa.

Let us discuss the results of studies with starch polysaccharides in more detail. Rheological measurement data indicate that the complex modulus of the composition with potato starch (Experiment 8, Table 4) is approximately 30% higher than with corn starch (Experiment 9, Table 4). The difference in mechanical moduli can apparently be explained by the difference in the composition of the starches, given in Table 5 (data from work [52]). As known, natural starch consists of two types of polymers: linear amylose and branched amylopectin. The mass of amylopectin in potato starch is half that of corn starch, while the mass of linear amylose in potato starch, which determines the elastic properties of gels, is almost 9 times higher than the corresponding values for corn starch. Interestingly, despite the lower mass of potato amylopectin, its radius of gyration is larger than that of corn starch amylopectin. That is, potato amylopectin clusters are packed more loosely, so the characteristic geometric size is larger. This circumstance can explain the higher complex modulus value of the composition with potato starch. According to the literature data [53,54,55,56], the molecular weights of amylose and amylopectin of rice starch are close to those of corn starch.

Replacing corn starch with corn flour increases the complex modulus even further—by almost 80%. This increase in modulus can be explained by the fact that corn flour also contains protein, which forms additional crosslinks with starch under the action of paraform, thereby making the composition stronger. Furthermore, the flour contains microfibers of cellulose, which can also crosslink with polyacrylamide via paraform and, as our studies have shown [31], significantly enhance the structural and mechanical properties of the gels through reinforcement. It is important to note that the market price of corn flour is 2–3 times lower than that of corn starch.

Among the tested polyacrylamide/polysaccharide gels, arabinogalactan (Experiment 12, Table 4) showed the lowest values of elastic modulus and complex modulus, equal to 39.3 and 47.5 Pa, respectively; nevertheless, the rheological properties are quite acceptable for conducting workover and isolation operations in wells.

Comparing the results of rheological testing of polyacrylamide gels with polyacrylamide/polysaccharide gels, it can be noted that, in general, all indicators of hydrogels with two polymers (polyacrylamide + polysaccharide) exceed those of hydrogels with only one polymer—polyacrylamide. This is understandable, since the hydrogels containing a copolymer of polyacrylamide with polysaccharides combine both the flexibility of the polyacrylamide component and the rigidity of the polysaccharide component. It should be noted that all rheological parameters of both polyacrylamide and polyacrylamide/polysaccharide gels are quite acceptable for water shutoff operations. Regarding polyacrylamide/polysaccharide gels, an expected trend of increasing mechanical strength with increasing molecular weight of the polysaccharide can be observed. For instance, guar gum has the highest molecular weight among all polysaccharides studied, and it shows the highest values of rheological parameters.

Comparing the results obtained in this work with our previous studies [31,57,58], it can be noted that the complex modulus of polyacrylamide/polysaccharide gels significantly exceeds that of polyacrylamide gels reinforced with nanoparticles and fibrous components. For example, hydrogels of polyacrylamide crosslinked with guar gum, corn flour, and rice flour have complex moduli of 106.2, 89.2, and 69.1 Pa, respectively. In contrast, hydrogels of polyacrylamide reinforced with carbon nanoparticles, nanosilica, and fibrous components have complex moduli of 33.0, 64.5, and 53.5 Pa, respectively. Thus, hydrogels with two crosslinked polymers outperform hydrogels with only polyacrylamide, even when reinforced with nanoparticles and fibrous components.

The influence of thermo-saline aggression on a hydrogel of the following composition: polyacrylamide grade A523—1 g (1.9%), CMC grade 9V—0.055 g (0.1%), paraform—0.21 g (0.4%), fresh water—51.37 g (97.6%) was studied with mineralized water (mineralization—273 g/L). For this purpose, an equal volume of mineralized water was poured into a vial containing the matured gel and thermostated for 24 h. The gel absorbed half the volume of mineralized water. Visual inspection showed that the gel became somewhat more mobile. Specifically, before the thermo-saline exposure, the hydrogel only shifted slightly when the vial was inverted (Figure S6a), whereas afterward, it exhibited a “tongue” (Figure S6b). This result of the thermo-saline resistance test is quite expected, since gels formed by covalent bonds do not change their structure depending on the ionic strength of the solution. This is their advantage over gels formed via ion-coordination bonds.

Concluding the experiments with polyacrylamide/polysaccharide gels, we managed to obtain a more environmentally friendly gel by excluding paraform from the component composition. In particular, a gel formed from polyacrylamide grade A523 (1.9%), xanthan (0.3%), hexamethylenetetramine (0.8%), and sulfamic acid (0.4%) had the following rheological indicators: elastic modulus 38.7 Pa, viscous modulus 26.7 Pa, and complex modulus 47.0 Pa. This gel is noticeably inferior in structural and mechanical properties to the gel crosslinked with paraform; however, it has quite acceptable properties and a higher degree of environmental friendliness.

**Figure 10:**
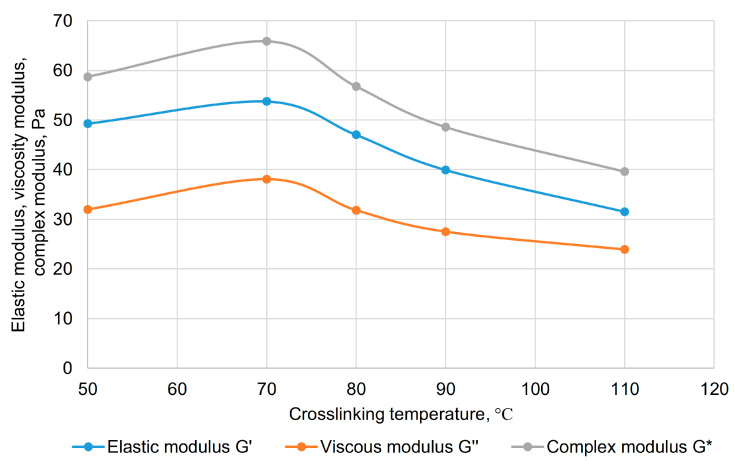
Results of oscillatory tests for the hydrogel crosslinked at different temperatures.

Given that obtaining polyacrylamide/polysaccharide gels through their crosslinking with paraform is a novel synthesis method for these copolymers, we conducted a sensitivity analysis of the reaction across a wide temperature range, at different component ratios, determining the viscoelastic properties of the gels, including under various pressures. The polyacrylamide/xanthan/paraform system was selected as the object of study. It was found that gelation of Composition 2 (Table 4) begins at 50 °C, while at 120 °C, the gel starts to degrade, becomes brittle, and a characteristic odor of formaldehyde is detected upon removal from the autoclave. When the reaction temperature was decreased to 80, 70, and 50 °C, the gelation time increased to 6, 6.5, and 12 h, respectively. At 40 °C, the composition did not crosslink within the 24 h observation period. Considering the gelation time and gel properties, the operational temperature range can be defined as 70–110 °C. A study of the rheological properties of gels obtained at different temperatures showed that at 70 °C, the highest values of the elastic modulus, viscous modulus, and complex modulus are observed (Figure 10).

**Figure 11:**
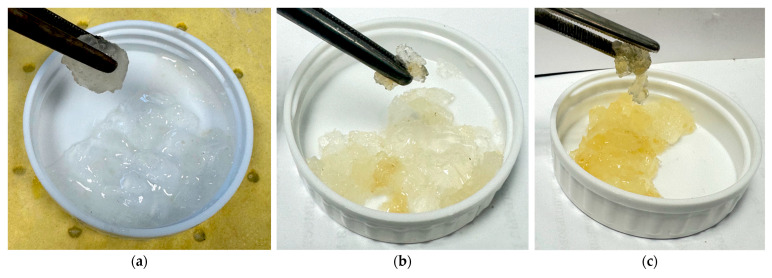
Appearance of hydrogel samples crosslinked at different temperatures: (**a**) 120 °C; (**b**) 130 °C; (**c**) 140 °C.

With a further temperature increase (above 70 °C), all rheological parameters decrease but remain quite acceptable for water shutoff materials. This dependence of gel properties on their synthesis temperature is explained by the following reasons: in the 50–70 °C range, the structural-mechanical properties increase due to the realization of a greater number of crosslinks, i.e., due to the network density in the gel. The subsequent decrease in all rheological parameters with a further temperature increase is associated with the weakening of hydrogen bonds, for which temperature increase is critical. Concurrently, the solvation shell of the crosslinked macromolecules thins, and the viscoelastic property indicators decrease. It should be noted that up to 110 °C, the properties of the obtained gels are fully sufficient to withstand the hydrodynamic pressure of water during well flowback after workover. Starting from 120 °C, the gel becomes brittle, breaking into separate pieces (Figure 11); however, gel syneresis did not occur up to 140 °C, as the copolymer did not form a separate condensed phase and did not release a free aqueous phase. This observation raises hope for the possibility of strengthening the gel with silica nanoparticles and maintaining the functionality of the nanocomposite at extreme temperatures.

**Figure 12:**
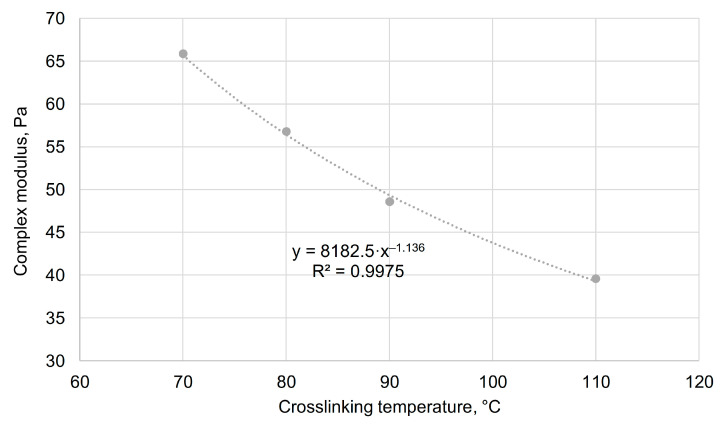
Regression dependence of the gel complex modulus on temperature in the range of 70–110 °C.

For the complex modulus (G*, Pa) in the temperature range from 70 to 110 °C, the following correlational dependence on temperature (t, °C) was obtained (Figure 12).

G* = 8182.5 · t^−1.136^. (1)

The obtained dependence will improve the accuracy of adapting the water shutoff composition injection technology for specific geological and physical conditions. If other polysaccharides are used in the gel-forming composition, the general form of the function will be preserved, but the coefficients in the equation will be different.

**Figure 13:**
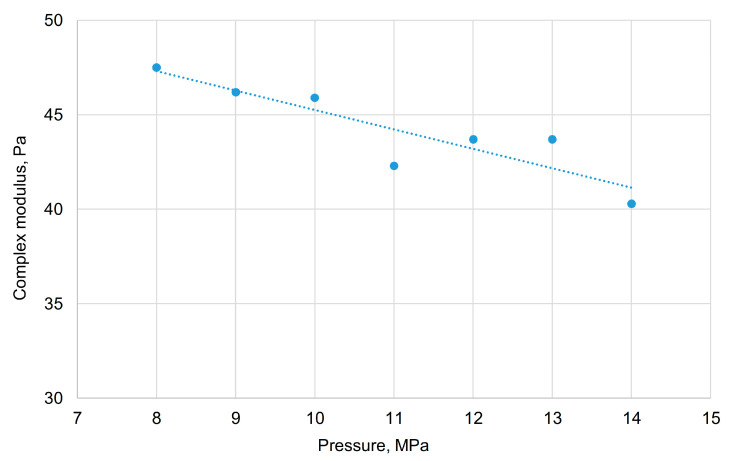
Dependence of the gel complex modulus on pressure in the range of 8–14 MPa.

Studies of the properties of gels aged under various pressures show that in the pressure range from 8 to 14 MPa, a slight decrease in the main parameters (elastic, viscous, and complex moduli) occurs. For instance, the complex modulus changes from 47.5 to 37.8 Pa (Figure 13), compared to 48.5 Pa for the baseline composition (90 °C, 0.1 MPa).

Varying the ratio of components in the polyacrylamide/xanthan/paraform system is limited from above by the polymer content. In Composition 1 (Table 4), with a content of both polyacrylamide and xanthan at 1.9%, an excessively high gelant viscosity is observed. Therefore, the content of both polymers was reduced relative to Composition 2 (Table 4). Variations in polymer concentrations of ± 0.2% did not lead to significant changes in the properties of the resulting hydrogels (Table 6).

All rheological parameters are approximately at the same level. A decrease in polyacrylamide concentration to 1.3% with a simultaneous increase in xanthan content to 0.7% already led to a noticeable decrease in the viscoelastic indicators of the hydrogel, but a result with an elastic modulus of 28.3 Pa and a complex modulus of 40.6 Pa is quite acceptable for a water shutoff material. The value of polyacrylamide content in the gel-forming composition should be considered the acceptable lower concentration limit.

Experiments 2 and 6–10 (Table 6) were performed to analyze the sensitivity of the composition to changes in the concentrations of the polymer components and the crosslinker. A decrease in PAM concentration to 1.7% (Experiment 2) led to a decrease in the complex modulus to 44.1 Pa, while an increase in concentration to 2.1% (Experiment 10) led to an increase in the complex modulus to 63.8 Pa. The obtained result correlates with the exponential dependence of polymer solution viscosity on PAM concentration. A decrease in xanthan concentration to 0.2% (Experiment 6) led to an insignificant change (within the margin of error) in the complex modulus to 49.8 Pa, while an increase in concentration to 0.4% (Experiment 7) led to an increase in the complex modulus to 58.6 Pa. A decrease in paraform concentration to 0.3% (Experiment 8) led to a decrease in the complex modulus to 45.6 Pa, while an increase in concentration to 0.5% (Experiment 9) led to an increase in the complex modulus to 55.2 Pa.

Based on the results of the sensitivity analysis, linear approximations of the obtained dependencies of the gel complex modulus on the varying factors were plotted in coordinates of relative changes (Figure S7).

**Figure 14:**
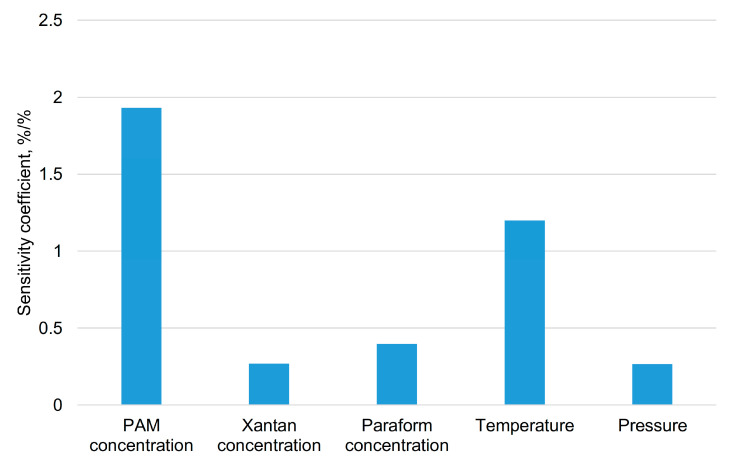
Absolute values of the sensitivity coefficients of the gel complex modulus to changes in the concentrations of polymers, crosslinker, temperature, and pressure.

The slope coefficients of the obtained dependencies characterize the sensitivity of the gel to changes in the factors (sensitivity coefficient). In Figure 14, these values are presented as a bar chart.

From Figure 14, it is evident that the change in PAM concentration has the greatest influence; for instance, when it changes by 1%, the gel complex modulus changes by 1.9%. Temperature also significantly affects the complex modulus—a 1% change in temperature leads to a 1.25% change in the gel complex modulus. Overall, the sensitivity analysis of the input parameters and conditions confirmed the reliability of the obtained results and identified the most significant factors.

Thus, all the obtained polyacrylamide/polysaccharide gels have high structural and mechanical properties, a comfortable gelation time, and an effective viscosity at 100 s^−1^ and can be successfully used for water shutoff in wells in high-temperature formations.

## 3. Conclusions

As a result of this work, we have developed gel-forming systems based on polyacrylamide (6 compositions) and polyacrylamide/polysaccharide copolymers (12 compositions) crosslinked with paraform, intended for well water shutoff in high-temperature formations. All studied hydrogels significantly surpass the baseline gel in their structural and mechanical properties and feature acceptable gelation times and gelant viscosities. The gels based on polyacrylamide with the selected crosslinkers exhibit an elastic modulus in the range of 27.9—46.3 Pa compared to 17.2 Pa for the baseline gel. For gels based on polyacrylamide/polysaccharide copolymers, the improvement over the baseline gel is even more substantial: the elastic modulus reaches values ranging from 39.3 to 1134.9 Pa. It was established that polyacrylamide/guar gels demonstrate relatively low gelant viscosity (891 mPa·s at 100 s^−1^) while maintaining high mechanical strength (G′ approximately 90 Pa). The gelation time of the investigated compositions ranged from 3 to 7 h, which is relevant for conducting water shutoff operations in horizontal wells in high-temperature formations (90 °C).

Analysis of the spectral data for the hydrogel obtained directly in an NMR spectrometer ampoule in deuterium oxide established that the crosslinking of polyacrylamide with xanthan via paraform occurs between the amide group of polyacrylamide and the hydroxyl group of the polysaccharide. This conclusion was further confirmed by a model reaction of low-molecular-weight analogs followed by detailed analysis of the reaction products’ mass spectra.

A wide range of polyacrylamide and polyacrylamide/polysaccharide gels has been obtained, allowing for the selection of the most suitable option depending on the field operation location, taking into account project logistics and budget constraints.

The sensitivity analysis of the hydrogel rheological characteristics to temperature, pressure, and component concentrations performed in this work confirmed the correctness of the obtained results. It also demonstrated that the variation in PAM concentration has the most significant influence on the rheological properties of the PAM/xanthan/paraform gel; specifically, a 1% change in PAM concentration altered the gel’s complex modulus by 1.9%. Temperature also substantially affects the complex modulus, with a 1% change in temperature resulting in a 1.25% change in the complex modulus. Prior to conducting field operations for water shutoff, the gel-forming composition is always adapted to specific geological and physical conditions, and the treatment design is optimized for each well. The knowledge of the applicability criteria obtained through the sensitivity analysis will help to reduce the pre-project preparation time for field operations.

**Figure 15:**
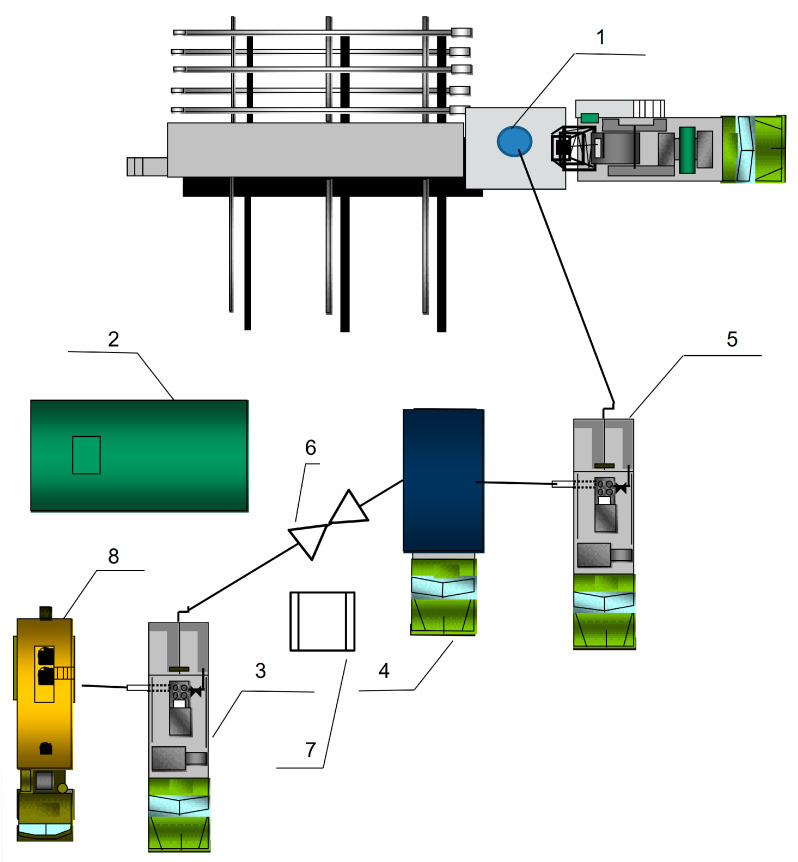
Schematic diagram of equipment layout for preparing gelant from the dry gel-forming composition and its injection (1—wellhead; 2—fresh water process tank; 3—cementing (pumping) unit CA-320; 4—blending and homogenization unit; 5—cementing (pumping) unit CA-320; 6—ejector; 7—pallet with dry commercial gel-forming composition (in 25 kg bags); 8—fresh water tank truck).

The developed hydrogels are designed for water isolation in granular reservoirs in deviated and horizontal wells. Since all components of the plugging compositions are dry substances, the most convenient form for application, without exception, is a commercial “single-bag” formulation; that is, all polymers and crosslinkers in the required proportions are pre-blended and poured into one bag. The bag is sealed and delivered directly to the well site for operations. Given that preparing the working solution simply involves dissolving the bag’s contents in water at the required concentration, the technology is simplified. Consequently, the number of process tanks and piping arrangements is minimized (Figure 15).

As can be seen from Figure 15, the following equipment is deployed at the wellhead (1): a fresh water tank truck (8), a cementing unit (3), an ejector (6), a blending and homogenization unit (4), and a cementing unit (5).

The dry mixture is fed via the ejector (6) into the blending and homogenization unit (4) using the cementing unit (3). Subsequently, the gelant solution is injected into the well (1) using the cementing unit (5).

Our future plans for developing this research direction involve studying the sensitivity of gel characteristics to variations in gelation temperature and component concentrations. Particular attention will be paid to investigating the long-term thermal degradation of the obtained gels and enhancing their stability through the introduction of nanoparticles (continuing our work [58]), as well as developing fibrous composites for well water shutoff in fractured reservoirs (continuing the research [31]).

## 4. Materials and Methods

### 4.1. Methodology

The research methodology stemmed from the work’s objectives, namely: to find simple methods for preparing polyacrylamide and polyacrylamide/polysaccharide gelants directly at the wellsite. Since we did not find any description in the literature of a method for crosslinking polyacrylamide with polysaccharides via paraform, the methodological approach involved conducting a model reaction with low-molecular-weight analogs, analyzing the structure of the resulting model reaction products, and subsequently carrying out the crosslinking of the high-molecular-weight components. The obtained results significantly simplified field operations. The “single-bag” commercial form of the reagents, where all composition components are pre-loaded in dry form into a bag, allows for simply dissolving the dry mixture in water at the field. After dissolution, the gelant is injected into the well. The development of the compositions involving polymers and crosslinker was conducted using methods of oscillatory rheometry and gelant viscosity control. Furthermore, a comprehensive sensitivity analysis was performed to assess the robustness of the gel properties against variations in key input parameters such as temperature, pressure, and component concentrations.

### 4.2. Gels and Additives

The following materials were used to prepare polyacrylamide or polyacrylamide/polysaccharide hydrogels: polyacrylamide grade A523 (LTD Anhui Tianrun Chemicals Co., Bengbu, China) with a molecular weight of 7.2 million Da and a hydrolysis degree of 22%; polyacrylamide grade EOR-1141 (TU 20.59.59-001-28618428-2018, LLC Khimintekh, Perm, Russia) with a molecular weight of 7.8 million Da and a hydrolysis degree of 24%; guar gum PTWG 7000F (LLC NIKA-PETROTEK, Yekaterinburg, Russia); xanthan gum (ETS Group, St. Petersburg, Russia); carboxymethyl starch grade Politsell CMS-bur 1 m. H and grade Politsell CMS-bur 1 m. V (TU 2262-016-32957739-2007, JSC Politsell, Vladimir, Russia); carboxymethyl cellulose grade 9V (TU 2231-017-32957739-2009, JSC “Politsell”, Vladimir, Russia); arabinogalactan (TU 9325-008-7069252-08 with amendment No. 2, JSC Ametis, Blagoveshchensk, Amur Region, Russia); rice flour, crushed corn grain, and corn starch (LLC Garnets, Vladimir, Russia).

The composition of starches of different origins supplied by LLC “Garnets” is given in Table 7 (based on the relevant GOST standards and Technical Specifications). Potato starch was isolated from potatoes of the Rozara variety [59], and its composition was determined directly by our team. For corn and potato starches, fats, fiber, and several other substances were not detected.

It is known that when starch is dissolved in hot water after swelling and gelatinization, preserved starch granule shells, referred to in the literature as “ghosts,” are observed [60,61]. Dissolution is assumed to involve the entanglement of polysaccharide chains. The colloidal solutions obtained at this stage exhibit characteristic opalescence, and obtaining a clear solution requires higher temperatures or the use of a special hydroimpulse apparatus or hydrothermal conditions [62]. Previous studies have found that mechanical treatment of starches and starch-containing raw materials significantly accelerates the dissolution of starch granules, allowing clear solutions to be obtained at lower temperatures and in less time. To facilitate dissolution, starches and grains (flour) were subjected to shear mechanical treatment in Pulverisette 5 mills (FRITSCH GmbH, Idar-Oberstein, Germany) using agate drums and agate balls. The ball diameter was 30 mm, with 10 balls per drum. The grinding bowl volume was 500 mL, and the mass of corn grain and flour was 30 g. The rotation speed was 300 revolutions per minute, and the grinding time was 20 min. The corn grain was pre-crushed in a grain knife mill to an average grain size of 1–2 mm.

The following reagents were used as crosslinkers: technical-grade resorcinol, 1st-class MFMC 98.5% (OKP 2472110130, JSC Uralkhimplast, Nizhniy Tagil, Russia); highest grade hydroquinone (GOST 19627-74 with amendments No. 1-3, JSC Khimreaktivsnab, Ufa, Russia); pyrocatechol (CAS Number 120-80-9, Rhodia, La Défense, France); paraform (TU 20.14.61-044-00203803-2021, JSC Metafrax Chemicals, Gubakha, Perm Krai, Russia); technical powdered lignosulfonates (TU 2455-028-00279580-2014, JSC Solikamskbumprom, Solikamsk, Russia); reagent CSSL-2M (TU 20.59.59-031-04698227-2022, JSC Azimut, Ufa, Russia).

### 4.3. General Procedure for Polyacrylamide Gel Preparation

First, the crosslinker(s) were dissolved in water. Then, polyacrylamide was added to the solution. The mixture was stirred for one hour at room temperature until globules disappeared. Subsequently, the flask was placed in a thermostat at 90 °C, and gel formation was visually confirmed by the appearance of a characteristic “tongue”.

### 4.4. General Procedure for Polyacrylamide/Polysaccharide Gel Preparation

Paraform was dissolved in water, followed by the addition of the polysaccharide. Finally, polyacrylamide was added to the solution. The mixture was stirred for one hour at room temperature until globules disappeared. Then, the gelant sample was placed in a glass flask and thermostated at temperatures ranging from 50 to 90 °C. The gelation time was determined by the appearance of a characteristic “tongue,” after which the rheological parameters of the resulting gels were determined using oscillatory rheometry. To obtain gels at temperatures from 100 to 140 °C, finger-type autoclaves, described in detail in [58], were used. The thermostating time in the autoclave was set to five hours.

To determine the influence of pressure from 8.0 to 14.0 MPa on the rheological properties of a gel formed at 90 °C, a sample of the latter was placed in a thermostated piston cell and maintained at 90 °C for 8 h under the specified pressure. The appearance of the cell is shown in Figure S8. This cell is part of the SMP-FES-2R filtration setup, whose characteristics are described in detail in [58]. Subsequently, the rheological parameters of the gels were determined using oscillatory rheometry.

### 4.5. Model Reaction Procedure

The following reagents were used in the model experiment: D-(+)-glucose (PanReac AppliChem, Barcelona, Spain), acetamide (pure grade), formalin (pure grade), and D_2_O (Sigma-Aldrich, St. Louis, MO, USA).

Reagents were weighed using Acculab ATL-220d4-I (ACCULAB Group, New York, NY, USA) laboratory analytical balances with an absolute error of ±0.0002 g.

A quantity of 0.46 mL (5.5 mmol) of formalin (33% solution) was added to a mixture of 1 g (5.5 mmol) of D-(+)-glucose and 0.32 g (5.5 mmol) of acetamide under stirring. The reaction mixture was heated at 90 °C for 6 h. The composition and structures of the reaction products were determined using IR spectroscopy, ^13^C, ^1^H, and ^15^N NMR spectroscopy, and chromatography–mass spectrometry.

### 4.6. Hydrogel Preparation for NMR Spectroscopy

To 4.4138 g (97.4%) of D_2_O under stirring, 0.0181 g (0.4%) of paraform was added, followed by 0.0136 g (0.3%) of xanthan. After 10 min, without stopping stirring, 0.0861 g (1.9%) of polyacrylamide was added in small portions. After the addition of all components, stirring was continued for 30 min. The resulting solution was placed in an NMR ampoule. The liquid height in the ampoule was 5 cm. To remove air bubbles, the ampoule with the liquid was heated to 90 °C. Then, bubbles were removed from the liquid by gently tapping the ampoule wall. Crosslinking of polyacrylamide grade A523 and xanthan was carried out directly in the ampoule at 90 °C for 6 h. The resulting composition was studied using ^13^C, ^1^H NMR spectroscopy, and IR spectroscopy.

### 4.7. Spectral Methods

IR spectra were obtained by attenuated total reflectance (ATR) with a diamond crystal at a resolution of 4 cm^−1^ using an FT-805 Fourier transform spectrometer (Simex, Novosibirsk, Russia). Sample spectra were recorded in the wavenumber range of 300–4000 cm^−1^.

NMR spectra were recorded on a Bruker Avance-III 500 spectrometer (Bruker Biospin AG, Fällanden, Switzerland) operating at frequencies of 500.13 (^1^H), 125.47 (^13^C), and 50.58 MHz (^15^N) using a 5 mm PABBO probe with Z-gradient, and on a Q.One Instruments Quantum-I Plus pulse spectrometer (Q.One Instruments, Fitchburg, MA, USA) operating at frequencies of 399.85 (^1^H) and 100.54 MHz (^13^C) using a 5 mm broadband probe with Z-gradient. The sample temperature was maintained constant at 298K. Chemical shifts in ^13^C, ^1^H, and ^15^N NMR spectra are reported relative to the signal of the internal D_2_O standard.

Mass spectra were recorded on an Agilent LC/Q-TOF 6530 chromatograph-mass spectrometer (Agilent Technologies, Santa Clara, CA, USA) and on an LC-MS-2010EV liquid chromatograph-mass spectrometer (Shimadzu, Kyoto, Japan) operating in chemical ionization modes.

### 4.8. Rheological Studies

Oscillatory studies were conducted using a Rheotest RN5.1 rotational viscometer (Rheotest Medingen GmbH, Ottendorf-Okrilla, Germany) with a “plate-plate” measuring system at 24 °C. The measuring plate diameter was D = 36 mm, and the gap between plates was h = 1 mm (Figure S9).

Oscillatory measurements were performed with a shear stress (τ) sweep ranging from 0.1 Pa to the oscillation breakdown point at approximately τ = 200 Pa, at an oscillation frequency (ν) of 1 Hz. The main measured parameters were: the elastic modulus (G′), viscous modulus (G″), complex modulus (G*), the crossover point (the intersection of G′ and G″) corresponding to the yield stress, and the linear viscoelastic range (LVR—the region where the elastic modulus remains constant).

For the oscillatory testing, an aliquot of the matured gel was extracted from its container using a dosing syringe and placed into the viscometer measuring cell (Figure S9). A gap of 1.05 mm was initially set to allow the excess hydrogel sample to be trimmed using special tweezers. Subsequently, the gap was adjusted to the final 1 mm, and the measurements were initiated. After testing, the sample was removed, and the measuring elements were cleaned. For each hydrogel composition, measurements were repeated 10 times. The average value was recorded in the table. The calculated standard deviation was: for polyacrylamide gels—2.8–4.2 Pa (elastic modulus) and 1.7–2.1 Pa (viscous modulus); for polyacrylamide/polysaccharide gels—6.7–9.5 Pa (elastic modulus) and 5.7–7.1 Pa (viscous modulus).

The effective viscosity of the samples was determined using a Haake Viscotester iQ rotational viscometer (Thermo Fisher Scientific, Waltham, MA, USA). This equipment is a hardware-software complex consisting of a measuring device (rotational viscometer) with a digital interface and a personal computer equipped with software for controlling the measuring device and processing experimental data.

The viscosity of polymer solution compositions (gelants) was determined at a shear rate of 100 s^−1^ using a “cylinder-cylinder” CC25 DIN Ti sensing system (sample volume 16.1 mL, gap between cylinders 1.06 mm). The measurement time per point was 30 s; 10 points were measured, and the average value was calculated. The measurement temperature was 24 °C.

### 4.9. Sensitivity Analysis

To verify the reliability of the obtained results, a sensitivity analysis of key hydrogel characteristics (elastic modulus, viscous modulus, complex modulus) to variations in input conditions (pressure, temperature, component concentrations) was conducted using the polyacrylamide/xanthan/paraform gel system as an example. The “One-at-a-time” method was applied, whereby all input parameters were fixed at their baseline (nominal) values and only one parameter was varied within a selected range.

For the temperature sensitivity analysis, gel crosslinking was performed in the temperature range of 40–140 °C. The sensitivity of the gel’s rheological parameters to pressure was studied in a thermostated piston cell using samples of pre-matured gel formed at atmospheric pressure and 90 °C. This approach is linked to the field practice of isolation operations, where the gelant, prepared at the wellhead under atmospheric conditions, is injected into the formation. A series of experiments was conducted at different pressures (8–14 MPa) and 90 °C, with the crosslinked composition maintained under pressure for 8 h. An analysis of sensitivity to the chemical composition of the gel was also performed, varying the concentrations of the polymers (PAM ±0.2 wt.%, xanthan ±0.1 wt.%) and the crosslinker (paraform ±0.1 wt.%).

## Figures and Tables

**Table 1:** Rheological properties of polyacrylamide gels.

Experiment	Composition *		G′ (Elastic Modulus), Pa	G″ (Viscous Modulus), Pa	G* (Complex Modulus), Pa
	Component	Content, wt.%			
1 (baseline gel)	Polyacrylamide A523	1.70	17.2	10.8	20.3
	Paraform	0.15			
	Resorcinol	0.05			
	Sulfamic acid	0.20			
2	Polyacrylamide A523	1.70	35.4	26.7	44.3
	Paraform	0.15			
	Hydroquinone	0.05			
	Na_2_CO_3_	0.10			
3	Polyacrylamide EOR-1141	1.70	31.5	18.9	36.8
	Paraform	0.15			
	Lignosulfonate	0.05			
4	Polyacrylamide EOR-1141	1.7	27.9	15.1	31.8
	Paraform	0.3			
	Lignosulfonate	0.1			
	Na_2_CO_3_	0.1			
5	Polyacrylamide EOR-1141	1.7	30.1	16.7	34.4
	Paraform	0.5			
	Lignosulfonate	0.2			
	Na_2_CO_3_	0.1			
6	Polyacrylamide EOR-1141	1.7	33.6	19.9	39.1
	Paraform	0.5			
	CSSL	0.2			
	Na_2_CO_3_	0.1			
7	Polyacrylamide A523	1.9	46.3	31.2	55.8
	Paraform	0.4			

* Solvent: fresh water; Gelation temperature: 90 °C.

**Table 2:** Results of the rheological parameter determinations for the hydrogel over 6 replicates.

Replicate Number	G′ (Elastic Modulus), Pa	G″ (Viscous Modulus), Pa	G* (Complex Modulus), Pa	Crossover, Pa	LVR, Pa	Max Angle, °
1	41.0	26.1	48.5	72.6	38.8	327
2	20.9	16.0	26.4	28.8	22.1	226
3	24.4	18.1	30.4	31.4	24.0	135
4	26.9	20.2	33.6	33.5	23.8	146
5	27.6	21.4	34.9	32.4	23.8	146
6	26.2	19.4	32.6	32.8	26.1	148
Average after breakdown	25.2	19.0	31.6	31.8	23.9	

**Table 3:** Gelant formulation in D_2_O.

Component	Content, wt.%	Mass, g
Polyacrylamide A523	1.9	0.0861
Xanthan	0.3	0.0136
Paraform	0.4	0.0181
D_2_O	97.4	4.4138

**Table 4:** Rheological properties of polyacrylamide/polysaccharide gels.

Experiment	Composition *		G′ (Elastic Modulus), Pa	G″ (Viscous Modulus), Pa	G* (Complex Modulus), Pa	Gelant Viscosity at 100 s^−1^, mPa·s	Gelation Time, h
	Component	Content, wt.%					
1	Polyacrylamide A523	1.9	110.3	45.2	119.2	1128	4
	Xanthan	1.9					
	Paraform	1.6					
2	Polyacrylamide A523	1.9	39.9	27.5	48.5	705	5
	Xanthan	0.3					
	Paraform	0.4					
3	Polyacrylamide A523	1.9	1134.9	338.0	1184.2	2098	3
	Guar gum	1.9					
	Paraform	1.6					
4	Polyacrylamide A523	1.9	89.9	56.5	106.2	891	4
	Guar gum	0.3					
	Paraform	0.4					
5	Polyacrylamide A523	1.9	46.9	29.7	55.5	686	4
	CMC grade 9V	0.1					
	Paraform	0.4					
6	Polyacrylamide A523	1.9	43.5	30.0	52.9	698	5
	Polisell CMS-bur 1 m. H	0.2					
	Paraform	0.4					
7	Polyacrylamide A523	1.9	43.9	31.7	54.2	705	5
	Politsell CMS-bur 1 m. V	0.2					
	Paraform	0.4					
8	Polyacrylamide A523	1.9	56.3	34.6	66.1	692	6
	Potato starch m/a FRITSCH-40’	0.2					
	Paraform	0.4					
9	Polyacrylamide A523	1.9	41.0	28.1	49.7	717	6
	Corn starch m/a FRITSCH-40’	0.2					
	Paraform	0.4					
10	Polyacrylamide A523	1.9	76.5	45.9	89.2	696	6
	Corn flour m/a FRITSCH-40’	0.3					
	Paraform	0.4					
11	Polyacrylamide A523	1.9	57.2	38.7	69.1	691	6
	Rice flour m/a FRITSCH-40’	0.3					
	Paraform	0.4					
12	Polyacrylamide A523	1.9	39.3	26.8	47.5	685	7
	Arabinogalactan	0.2					
	Paraform	0.4					

* Solvent: fresh water; Gelation temperature: 90 °C.

**Table 5:** Molar mass (M) and radius of gyration (Rg) for the amylose and amylopectin.

Polysaccharides	Molecular Mass, M·10^−6^, Da [52]	Radius of Gyration, Rg, nm [52]
Amylose of maize starch	2.1	19.4
Amylose of potato starch	19.6	31.8
Amylopectin of maize starch	112.2	219.6
Amylopectin of potato starch	60.9	224.3

**Table 6:** Rheological properties of polyacrylamide/polysaccharide gels with varying concentrations of polymers and crosslinker.

Experiment	Composition *		G′ (Elastic Modulus), Pa	G″ (Viscous Modulus), Pa	G* (Complex Modulus), Pa	Crossover, Pa	LVR, Pa
	Component	Content, wt.%					
1	Polyacrylamide A523	1.9	39.9	27.5	48.5	53.2	22.3
	Xanthan	0.3					
	Paraform	0.4					
2	Polyacrylamide A523	1.7	35.6	26.0	44.1	49.0	23.6
	Xanthan	0.3					
	Paraform	0.4					
3	Polyacrylamide A523	1.7	38.3	26.3	46.6	51.5	24.6
	Xanthan	0.4					
	Paraform	0.4					
4	Polyacrylamide A523	1.5	34.2	24.0	41.8	47.9	27.9
	Xanthan	0.5					
	Paraform	0.4					
5	Polyacrylamide A523	1.3	28.3	21.5	35.6	40.6	28.2
	Xanthan	0.7					
	Paraform	0.4					
6	Polyacrylamide A523	1.9	40.5	29.0	49.8	59.3	31.6
	Xanthan	0.2					
	Paraform	0.4					
7	Polyacrylamide A523	1.9	49.5	31.4	58.6	67.3	33.9
	Xanthan	0.4					
	Paraform	0.4					
8	Polyacrylamide A523	1.9	37.2	26.3	45.6	53.7	29.3
	Xanthan	0.3					
	Paraform	0.3					
9	Polyacrylamide A523	1.9	45.7	31.0	55.2	71.2	44.1
	Xanthan	0.3					
	Paraform	0.5					
10	Polyacrylamide A523	2.1	53.9	34.1	63.8	81.5	49.8
	Xanthan	0.3					
	Paraform	0.4					

* Solvent: fresh water; Gelation temperature: 90 °C.

**Table 7:** Average composition per 100 g.

Raw Material	Carbohydrates, g	Protein, g	Fats, g	Fiber, g	Ash, g	Water, g
Rice flour	80	5	2	1	1	11
Crushed corn grain	71	9	4	3	1	12
Corn starch	85	1	n.d. ^1^	n.d.	n.d.	14
Potato starch, Rozara variety	83	n.d.	n.d.	n.d.	1	16

^1^ n.d.—not determined.

## Data Availability

The original contributions presented in this study are included in the article. Further inquiries can be directed to the corresponding authors.

## Supplementary Materials

The following supporting information can be downloaded at: https://www.mdpi.com/article/10.3390/gels11110862/s1, Figure S1: Sydansk gel test: (a) Thermal Exposure Time (TET) = 3 h, test tube in upright position; (b) TET = 3 h, test tube in inverted position; (c) TET = 4 h, test tube in upright position; (d) TET = 4 h, test tube in inverted position; (e) TET = 6 h, test tube in upright position; (f) TET = 6 h, test tube in inverted position; Figure S2: Successive stages of qualitative stretching and recovery tests of the polyacrylamide-paraform hydrogel filament: (a) Initial state (1st cycle); (b) Stretched state; (c) Initial state (5th cycle); (d) Stretched state (5th cycle); Figure S3: Mass spectrum (positive ion mode) of the reaction products of acetamide with D-glucose and formaldehyde; Figure S4: ^1^H NMR spectrum of the reaction products of acetamide with D-glucose and formaldehyde; Figure S5: {^1^H, ^15^N} HMBC NMR spectrum of the reaction products of acetamide with D-glucose and formaldehyde; Figure S6: Appearance of the inverted vial with hydrogel during thermo-saline exposure: (a) before; (b) after 24 h exposure to mineralized water (273 g/L); Figure S7: Dependencies of the relative change in complex modulus on the relative change in influencing factors; Figure S8: Photograph of the thermostated cell; Figure S9. Viscometer measuring cell: (a) Schematic diagram of the sensing elements of the rotational viscometer; (b) Photograph of the measuring cell.

## Abbreviations

The following abbreviations are used in this manuscript:

CSSL	Condensed sulfite spent liquor
NMR	Nuclear magnetic resonance
HMBC	Heteronuclear multiple bond correlation
APCI	Atmospheric pressure chemical ionization
IR	Infrared
FT-IR	Fourier-transform infrared spectroscopy
CMS	Carboxymethyl starch
CMC	Carboxymethyl cellulose
MFMC	Mass fraction of the main component
TET	Thermal exposure time
