# Supplementary material for: Polyacrylamide and Polyacrylamide/Polysaccharide Hydrogels for Well Water Shutoff in High-Temperature Reservoirs
Source: Gels. 2025 Oct 28;11(11):862. doi: 10.3390/gels11110862 (PMC12652023; doi:10.3390/gels11110862)
Supplement: Supplementary file 1 [file gels-11-00862-s001.zip › gels-3862933-supplementary.pdf]

Supplementary Material

Polyacrylamide and Polyacrylamide/Polysaccharide Hydrogels for Well Water Shutoff in High-Temperature Reservoirs

Aleksey Telin [1,*], Natalia Sergeeva [1], Rustem Asadullin [2], Ekaterina Gusarova [3], Ravil Yakubov [2,*], Vladimir Dokichev [4], Anatoly Politov [5], Elina Sunagatova [2], Natalia Gibadullina [4], Galina Teptereva [2] and Lyubov Lenchenkova [2]

[1] Ufa Scientific and Technical Center, LLC, 99/3, Kirova Street, 450078 Ufa, Russia
[2] Ufa State Petroleum Technological University, 1, Kosmonavtov Street, 450064 Ufa, Russia
[3] Interdisciplinary Research Laboratory of Oilfield Chemistry, Ufa University of Science and Technology, 12, Karla Marksa Street, 450008 Ufa, Russia
[4] Ufa Institute of Chemistry, Ufa Federal Research Center, Russian Academy of Sciences, 71, Oktyabrya Avenue, 450054 Ufa, Russia
[5] Institute of Solid State Chemistry and Mechanochemistry of Siberian Branch RAS; 630128, 18, Kutateladze Street, Novosibirsk, Russia

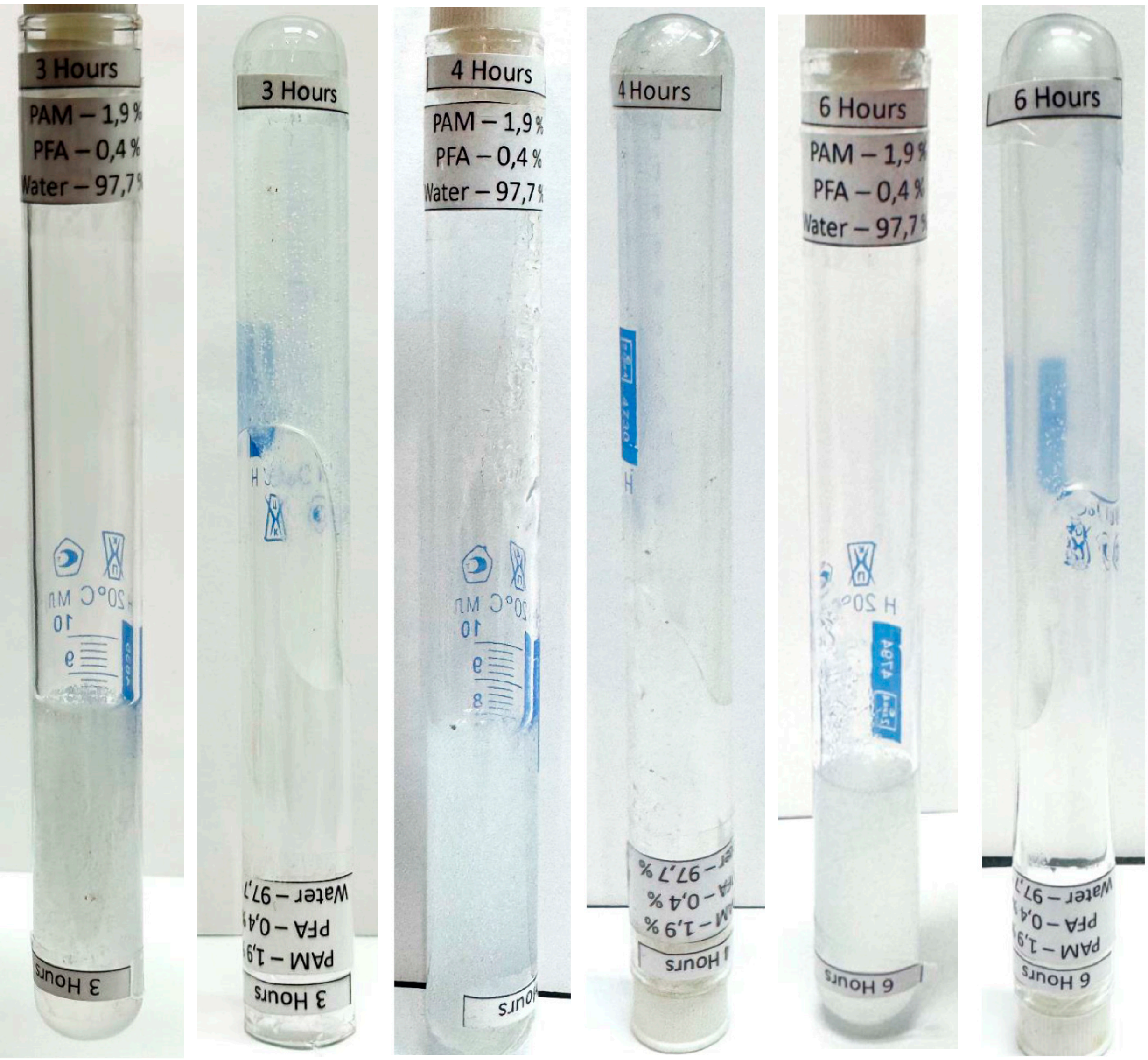

(**a**) (**b**) (**c**) (**d**) (**e**) (**f**)

Figure S1. Sydansk gel test: (a) Thermal Exposure Time (TET) = 3 hours, test tube in upright position; (b) TET = 3 hours, test tube in inverted position; (c) TET = 4 hours, test tube in upright position; (d) TET = 4 hours, test tube in inverted position; (e) TET = 6 hours, test tube in upright position; (f) TET = 6 hours, test tube in inverted position.

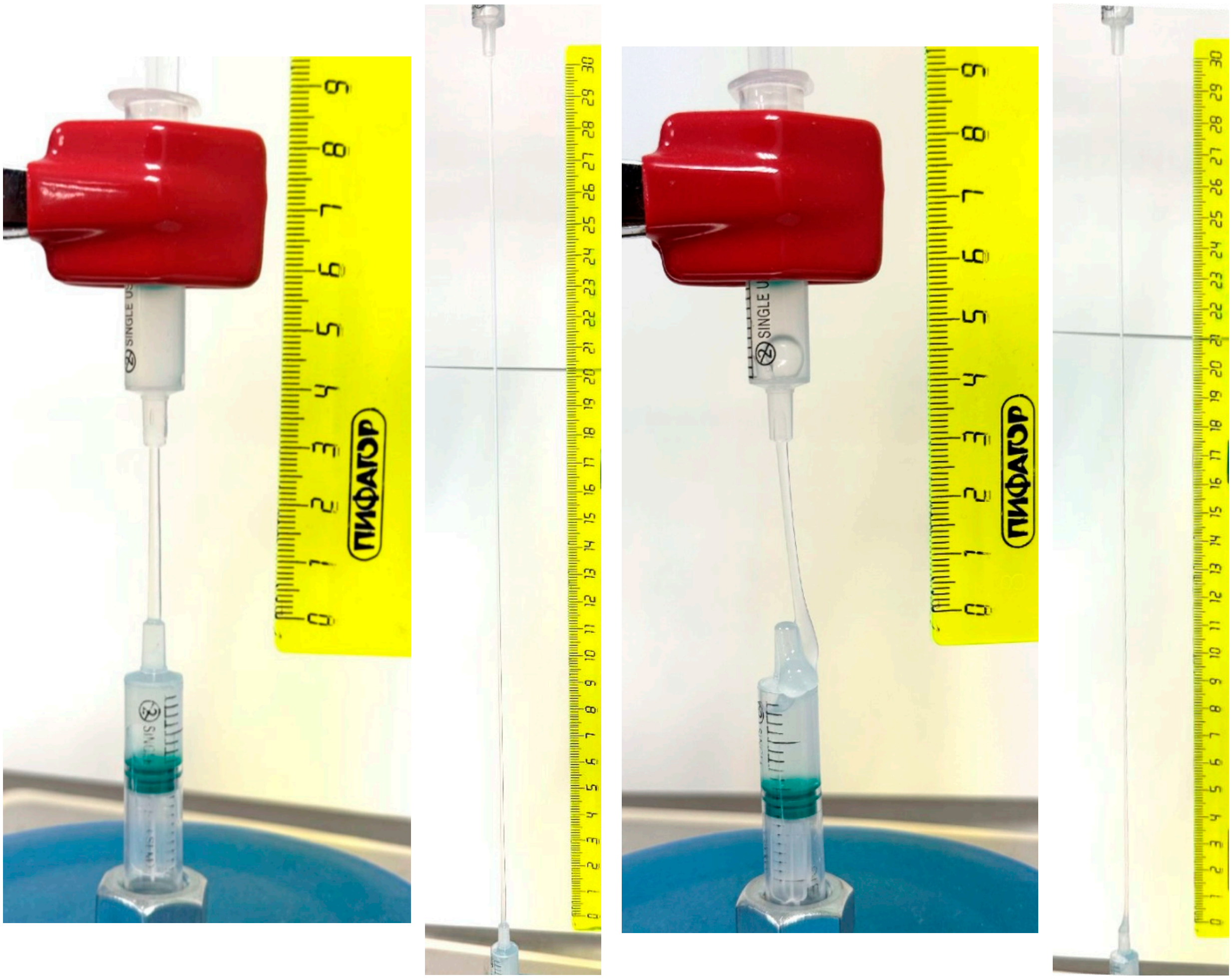

(**a**) (**b**) (**c**) (**d**)

Figure S2. Successive stages of qualitative stretching and recovery tests of the polyacrylamide-paraform hydrogel filament: (a) Initial state (1st cycle); (b) Stretched state; (c) Initial state (5th cycle); (d) Stretched state (5th cycle).

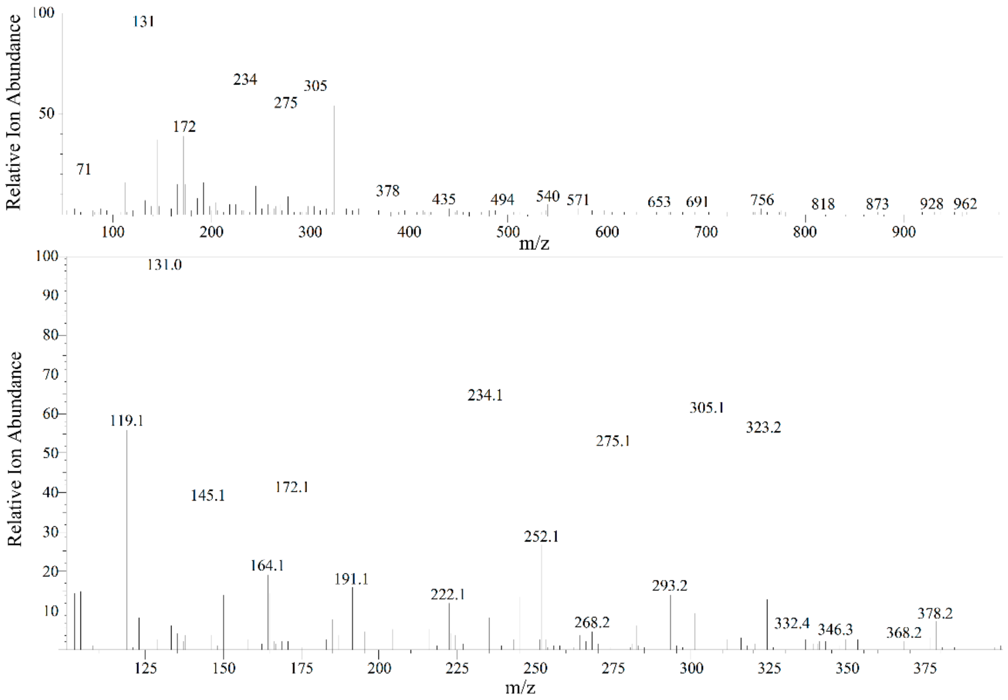

Figure S3. Mass spectrum (positive ion mode) of the reaction products of acetamide with D-glucose and formaldehyde.

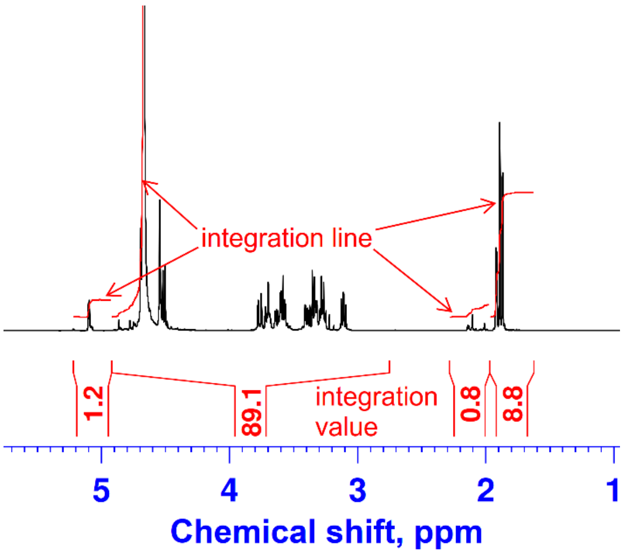

Figure S4. ^{1}H NMR spectrum of the reaction products of acetamide with D-glucose and formaldehyde.

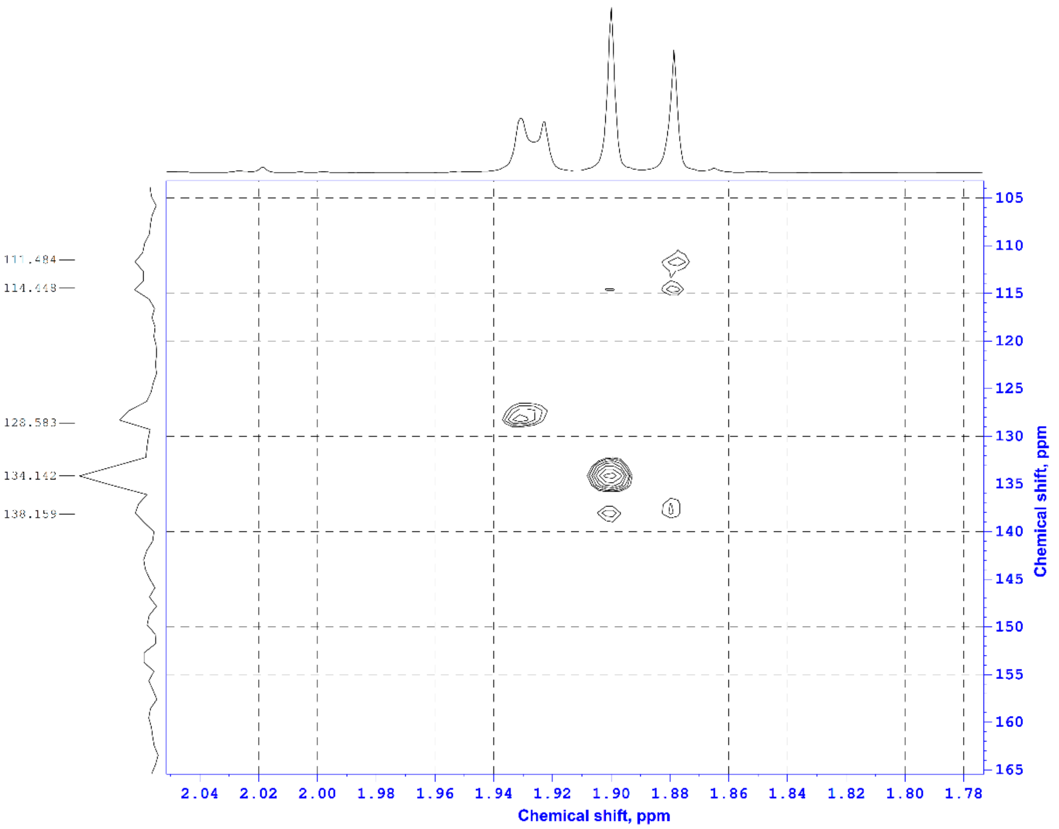

Figure S5. {^{1}H, ^{15}N} HMBC NMR spectrum of the reaction products of acetamide with D-glucose and formaldehyde.

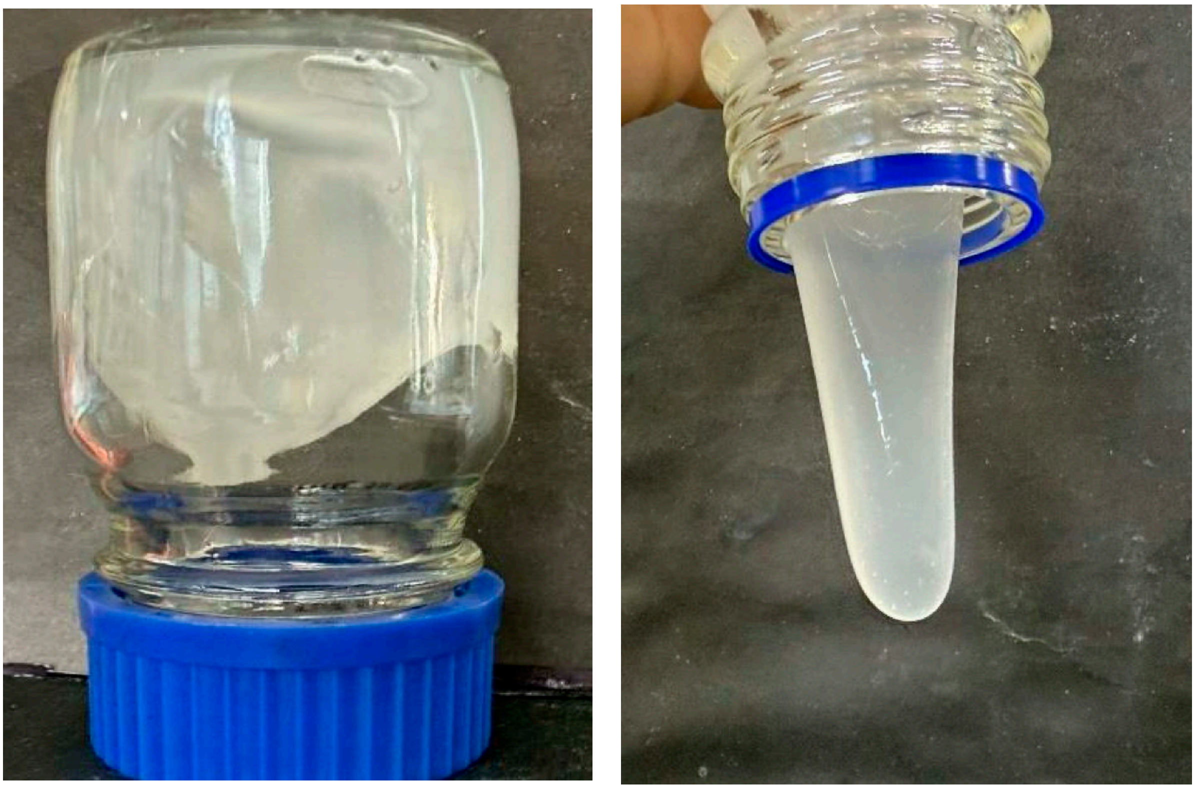

(**a**) (**b**)

Figure S6. Appearance of the inverted vial with hydrogel during thermo-saline exposure: (a) before; (b) after 24-hour exposure to mineralized water (273 g/L).

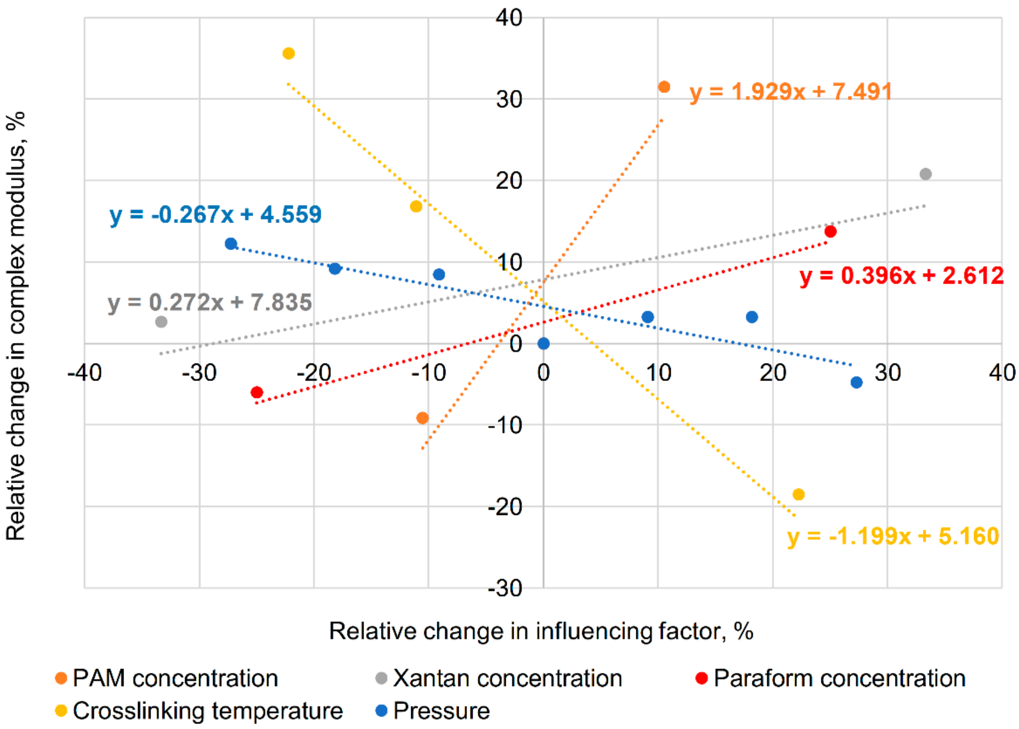

Figure S7. Dependencies of the relative change in complex modulus on the relative change in influencing factors.

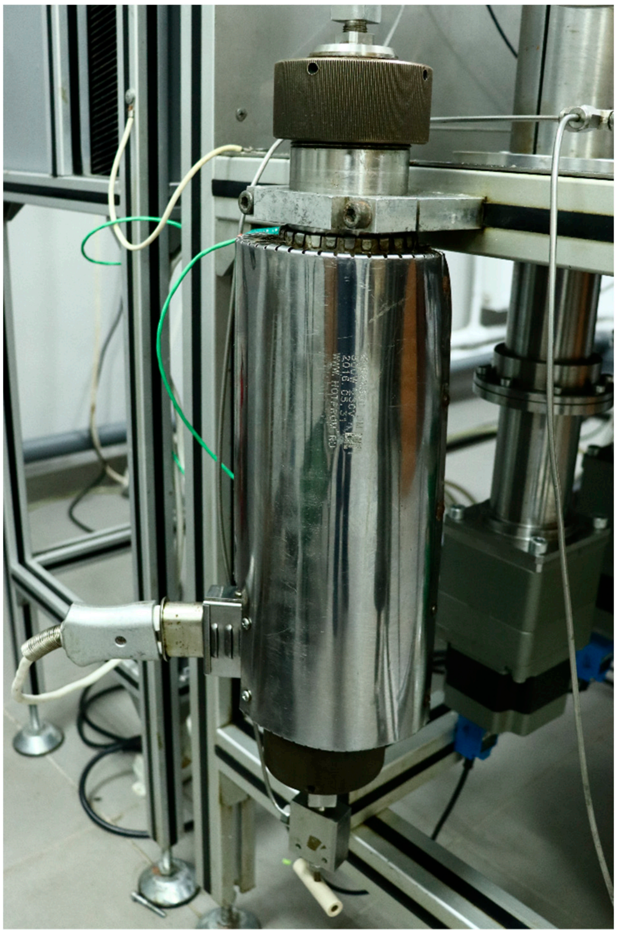

Figure S8. Photograph of the thermostated cell.

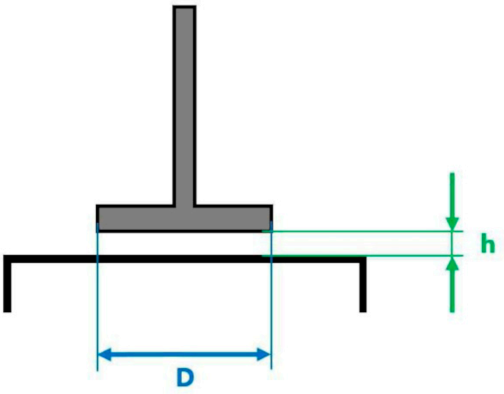

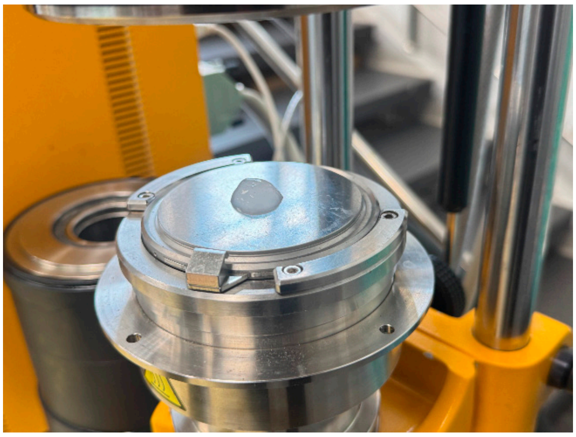

(**a**) (**b**)

Figure S9. Viscometer measuring cell: (a) Schematic diagram of the sensing elements of the rotational viscometer; (b) Photograph of the measuring cell.
