# Supplementary figures and images for: Polyacrylamide and Polyacrylamide/Polysaccharide Hydrogels for Well Water Shutoff in High-Temperature Reservoirs
Source: Gels. 2025 Oct 28;11(11):862. doi: 10.3390/gels11110862 (PMC12652023; doi:10.3390/gels11110862)

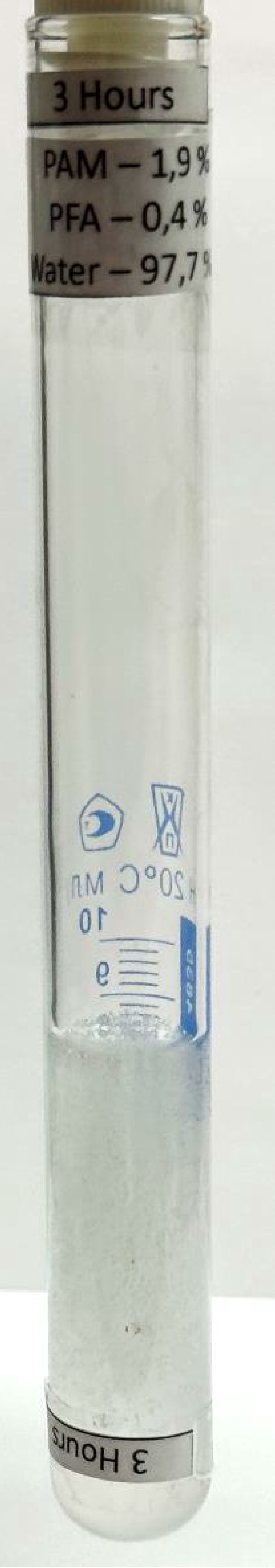

Supplement: Supplementary file 1 [file gels-11-00862-s001.zip › Figure_S1a.png]

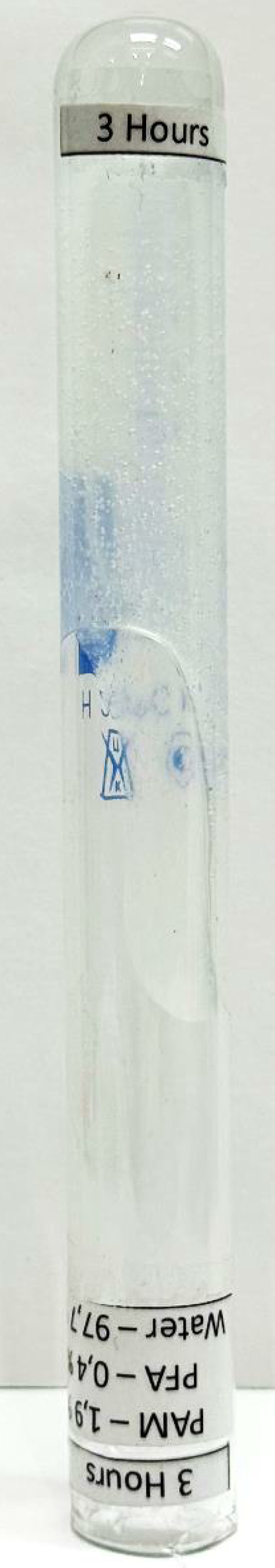

Supplement: Supplementary file 1 [file gels-11-00862-s001.zip › Figure_S1b.png]

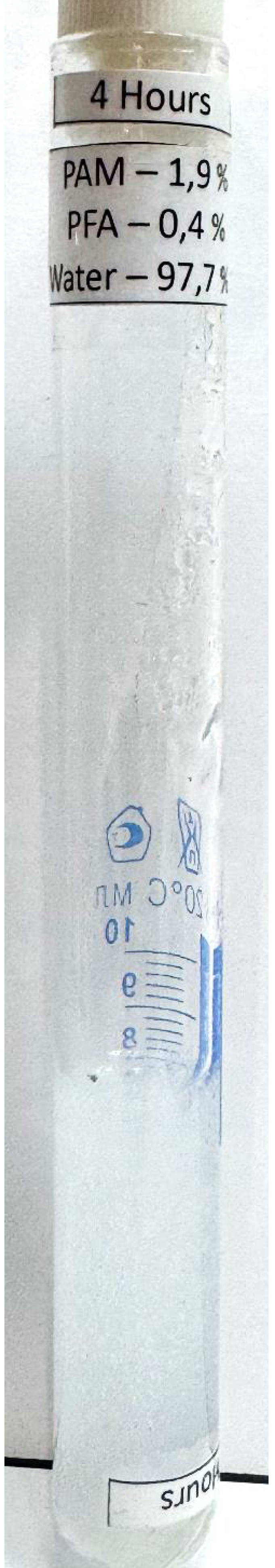

Supplement: Supplementary file 1 [file gels-11-00862-s001.zip › Figure_S1c.png]

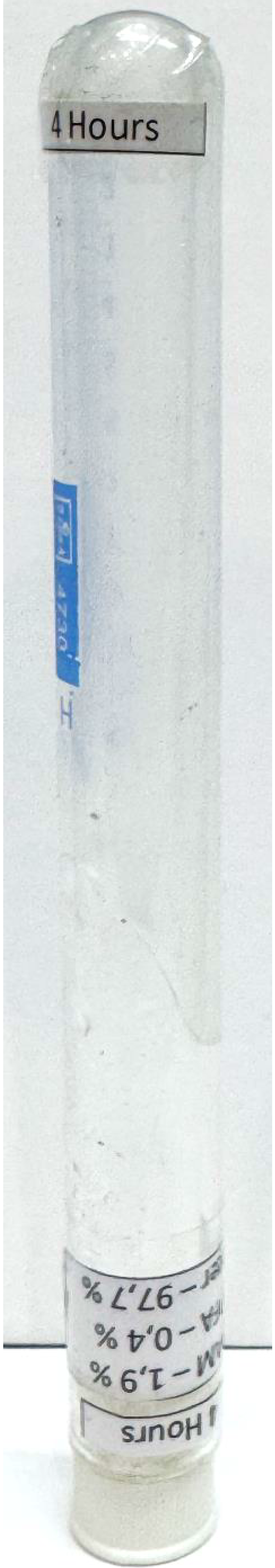

Supplement: Supplementary file 1 [file gels-11-00862-s001.zip › Figure_S1d.png]

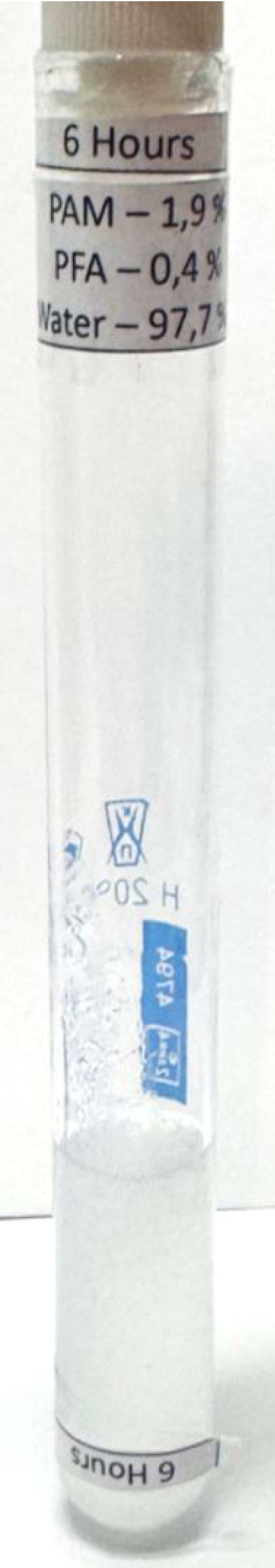

Supplement: Supplementary file 1 [file gels-11-00862-s001.zip › Figure_S1e.png]

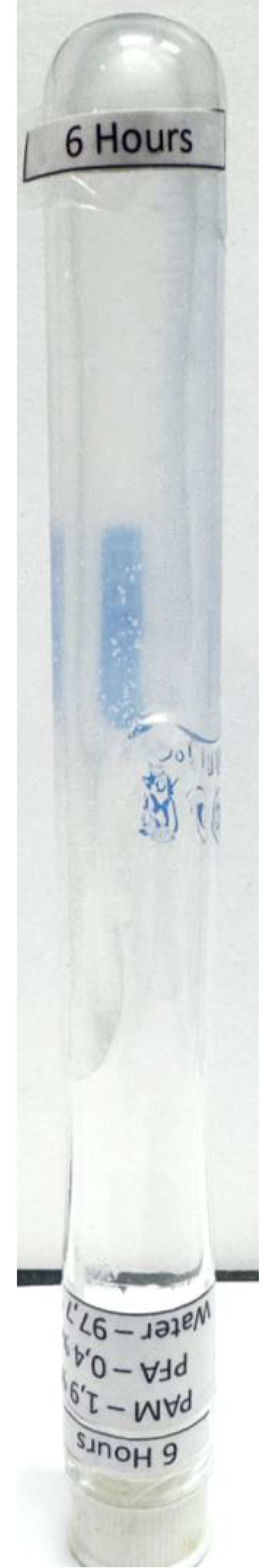

Supplement: Supplementary file 1 [file gels-11-00862-s001.zip › Figure_S1f.png]

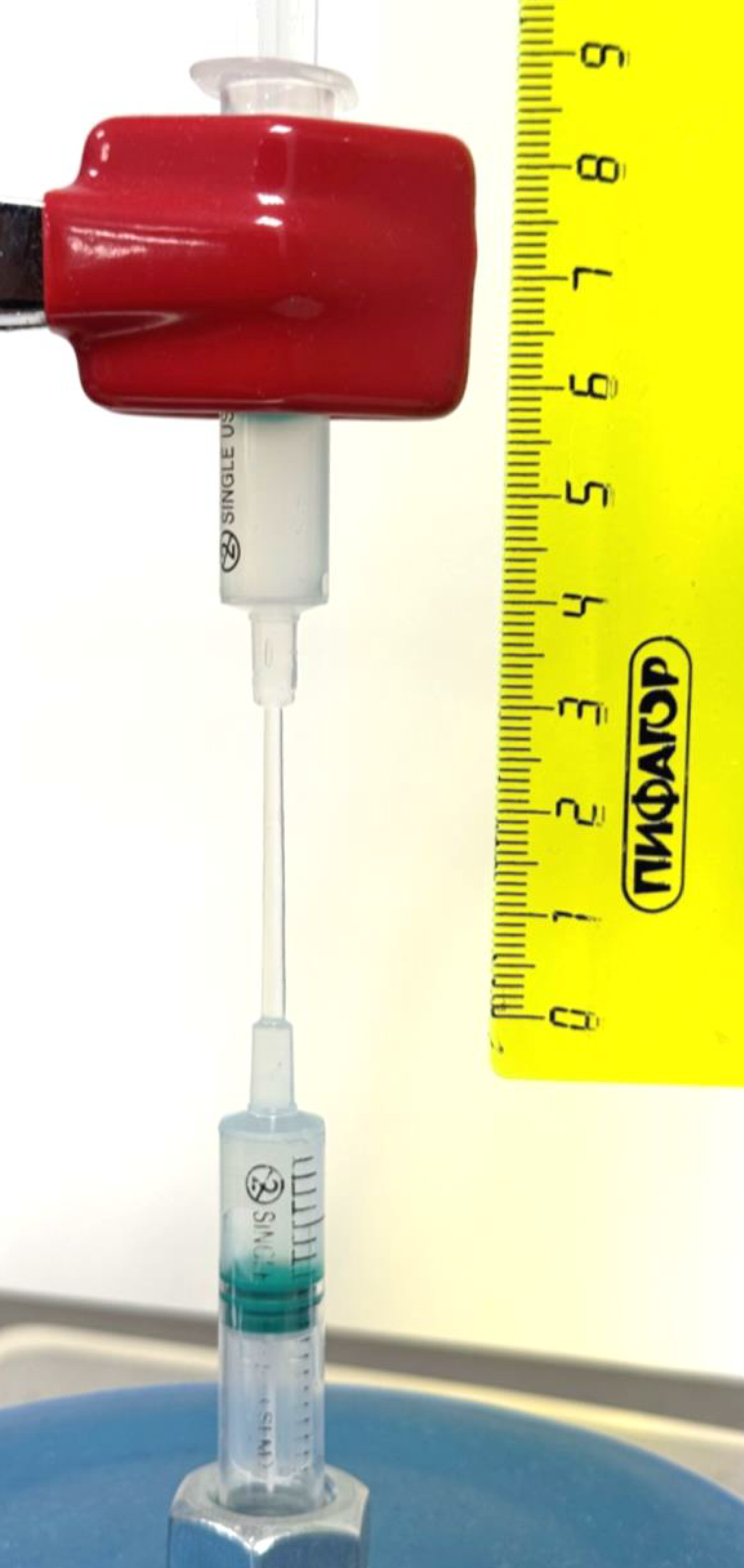

Supplement: Supplementary file 1 [file gels-11-00862-s001.zip › Figure_S2a.png]

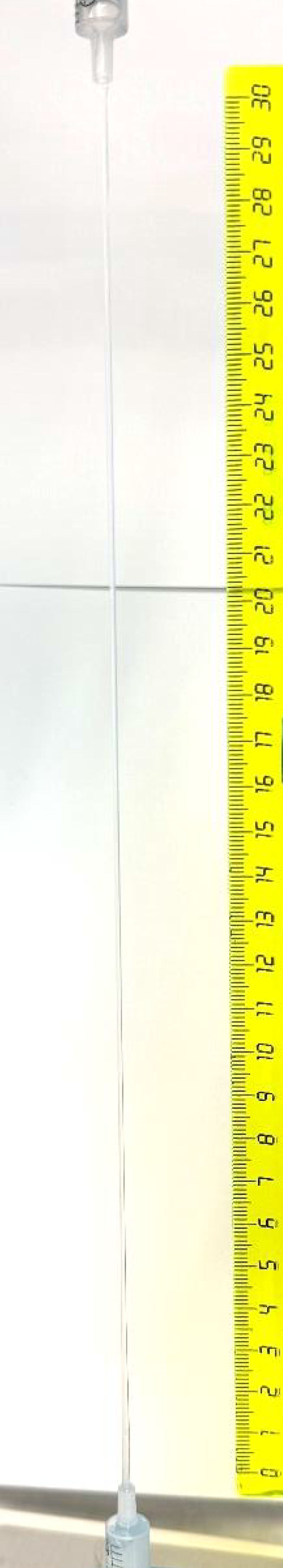

Supplement: Supplementary file 1 [file gels-11-00862-s001.zip › Figure_S2b.png]

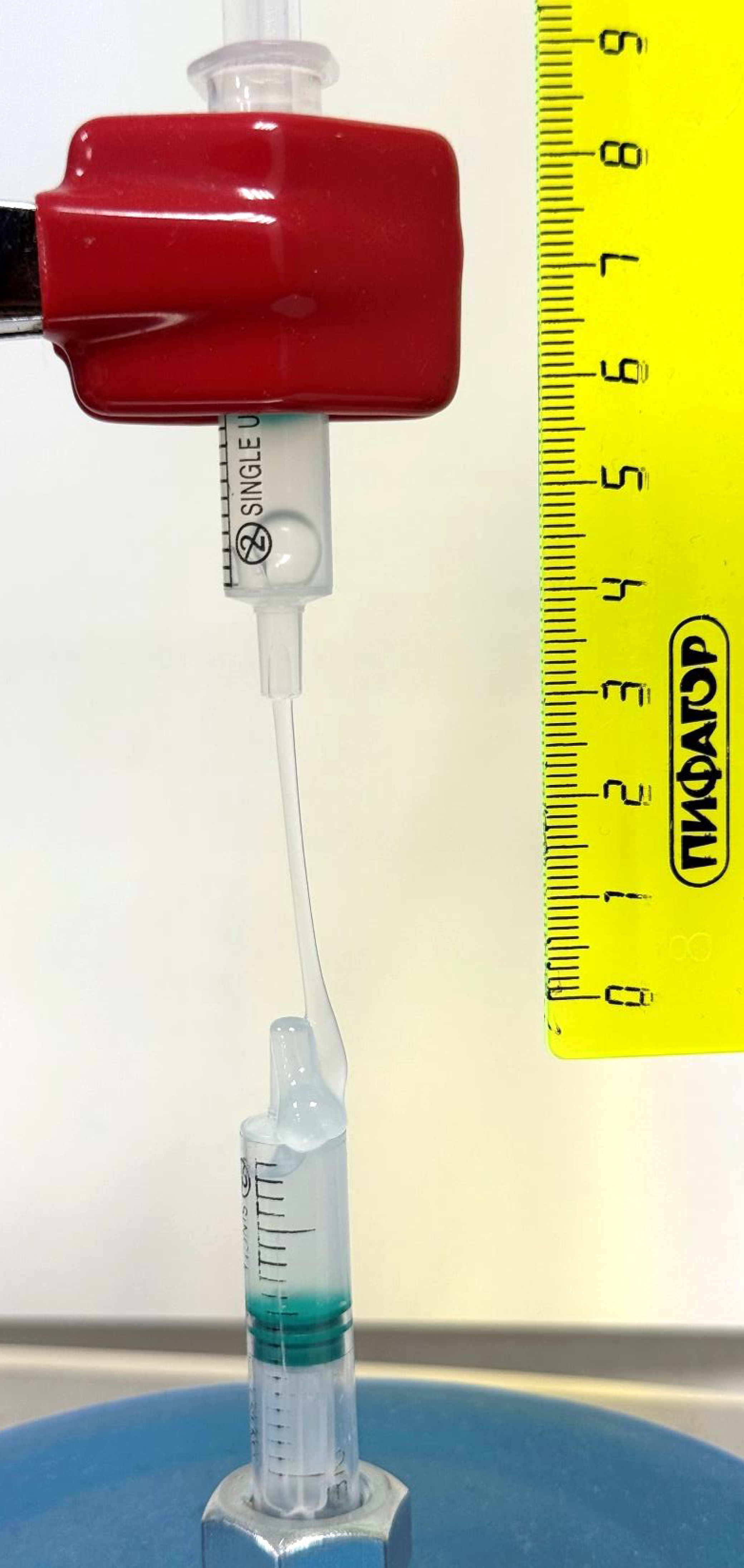

Supplement: Supplementary file 1 [file gels-11-00862-s001.zip › Figure_S2c.png]

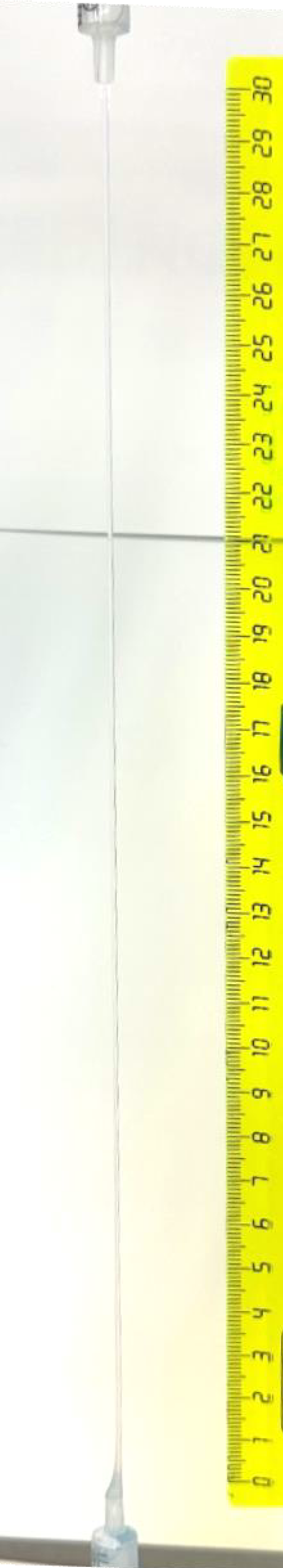

Supplement: Supplementary file 1 [file gels-11-00862-s001.zip › Figure_S2d.png]

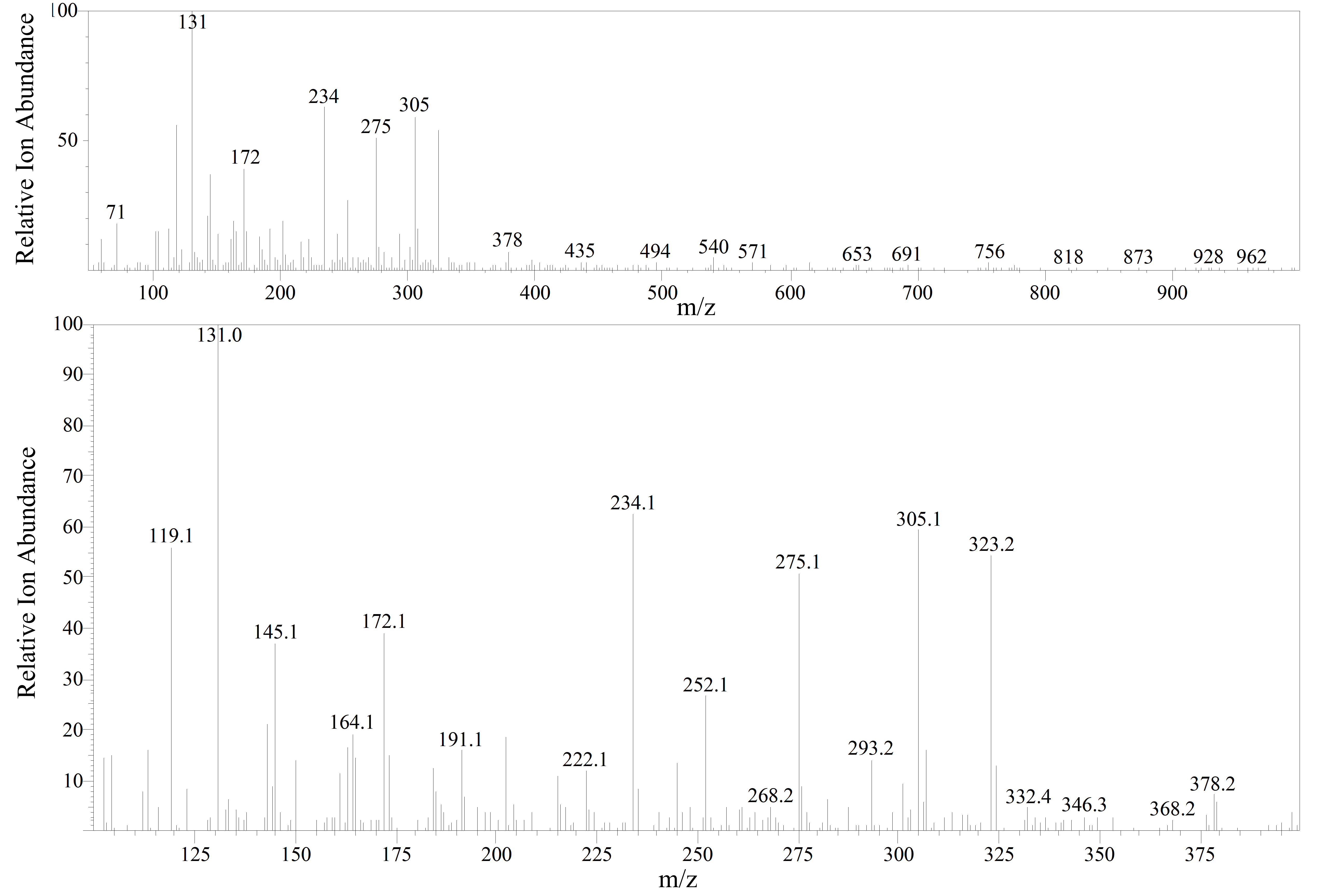

Supplement: Supplementary file 1 [file gels-11-00862-s001.zip › Figure_S3.png]

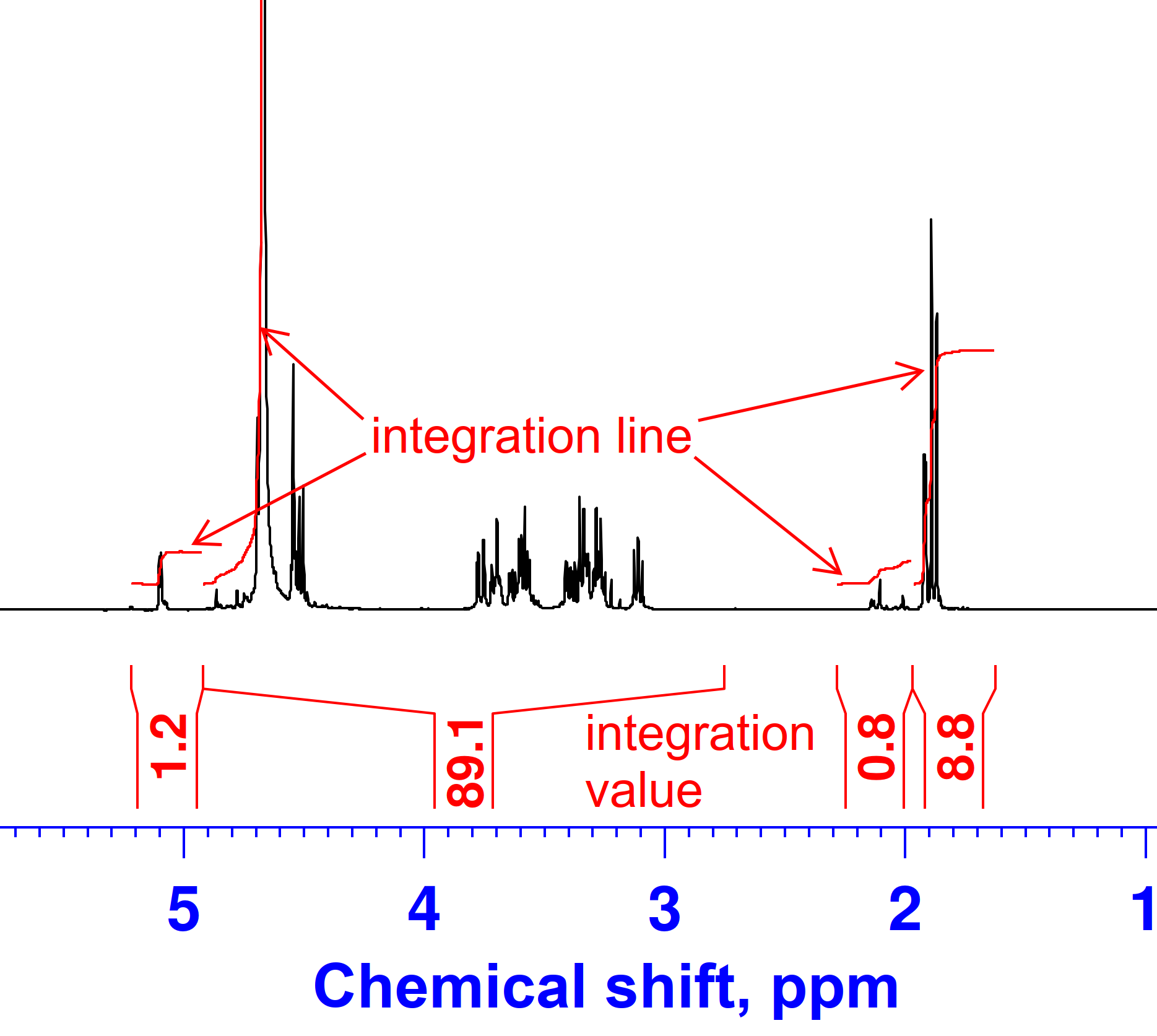

Supplement: Supplementary file 1 [file gels-11-00862-s001.zip › Figure_S4.png]

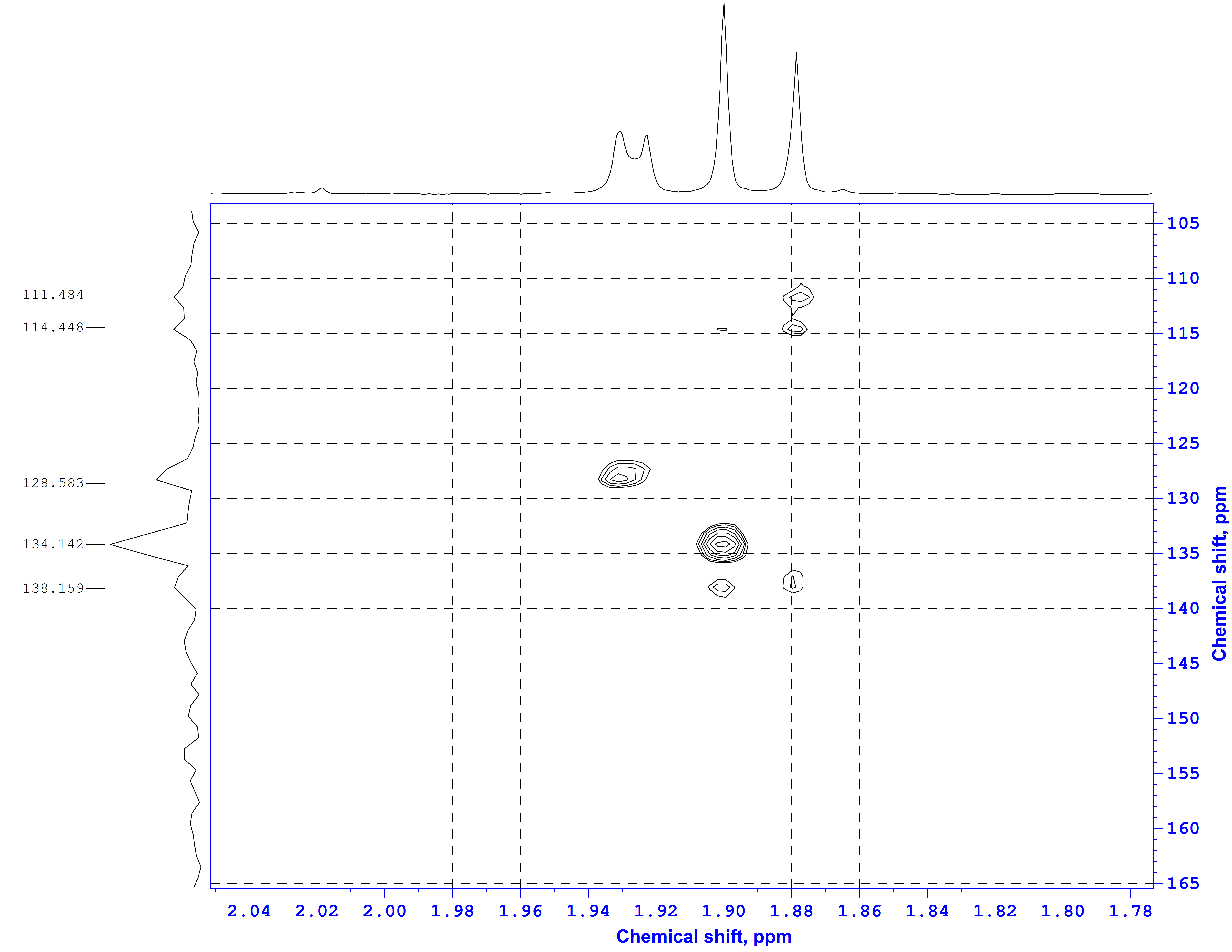

Supplement: Supplementary file 1 [file gels-11-00862-s001.zip › Figure_S5.png]

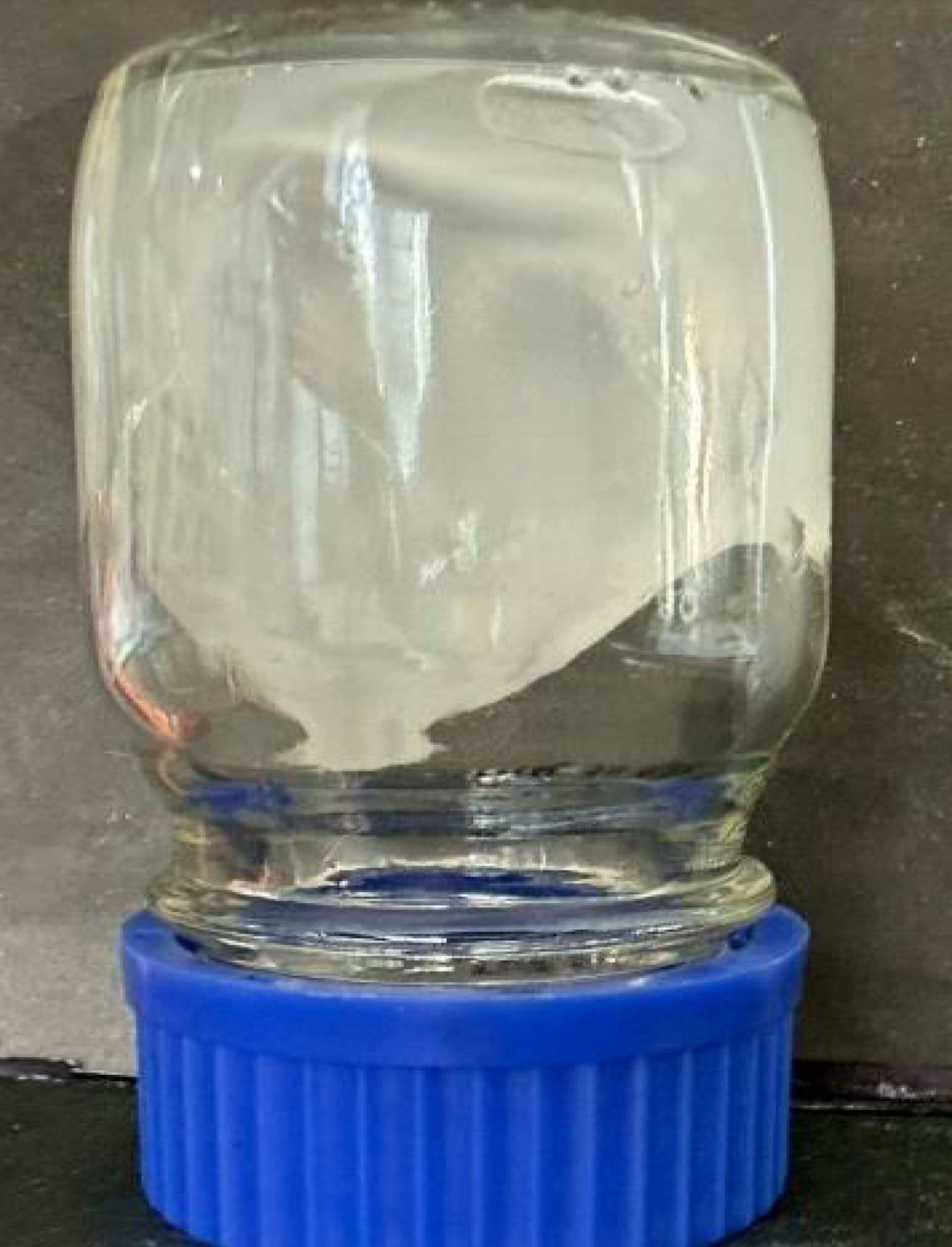

Supplement: Supplementary file 1 [file gels-11-00862-s001.zip › Figure_S6a.png]

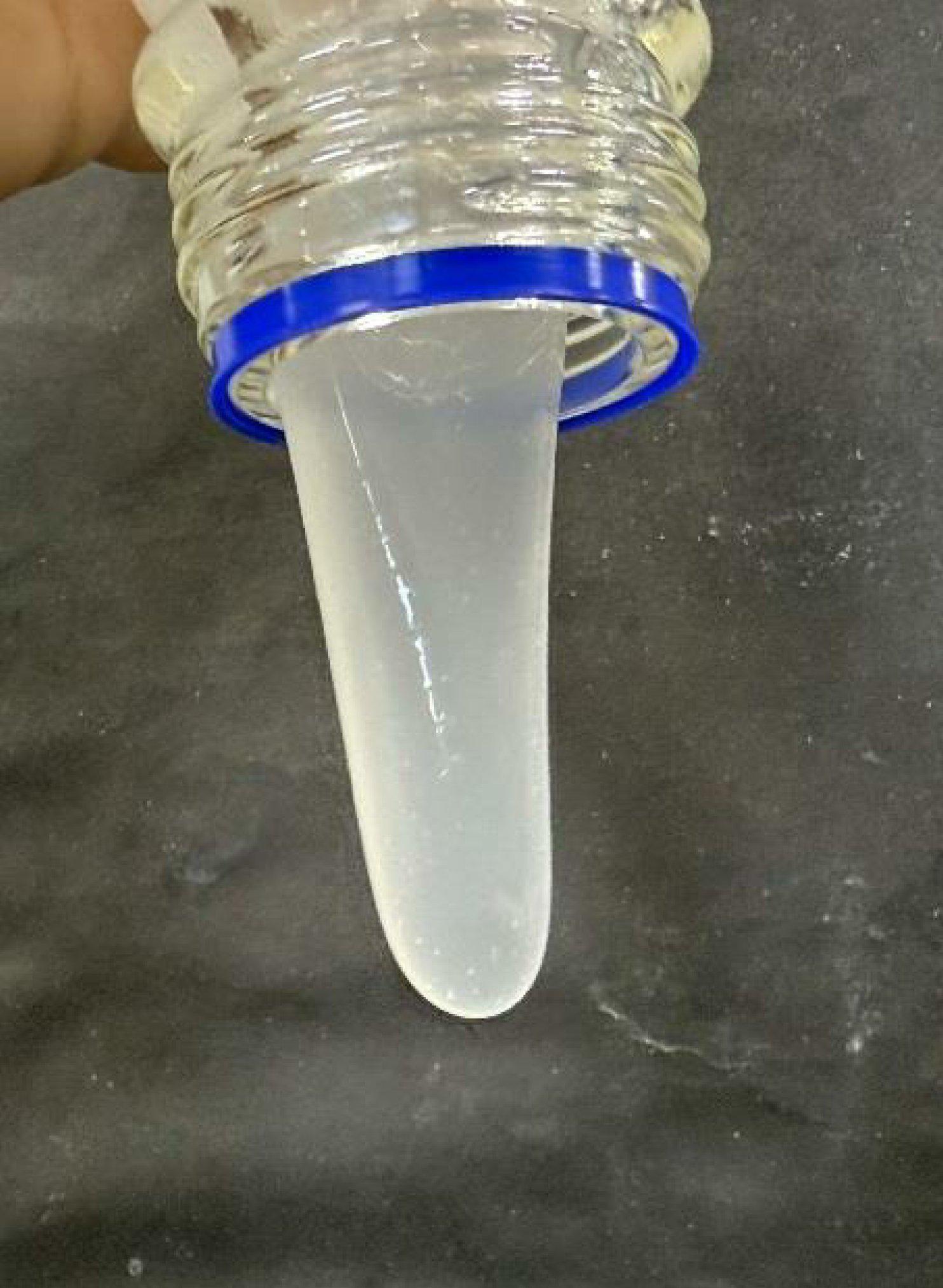

Supplement: Supplementary file 1 [file gels-11-00862-s001.zip › Figure_S6b.png]

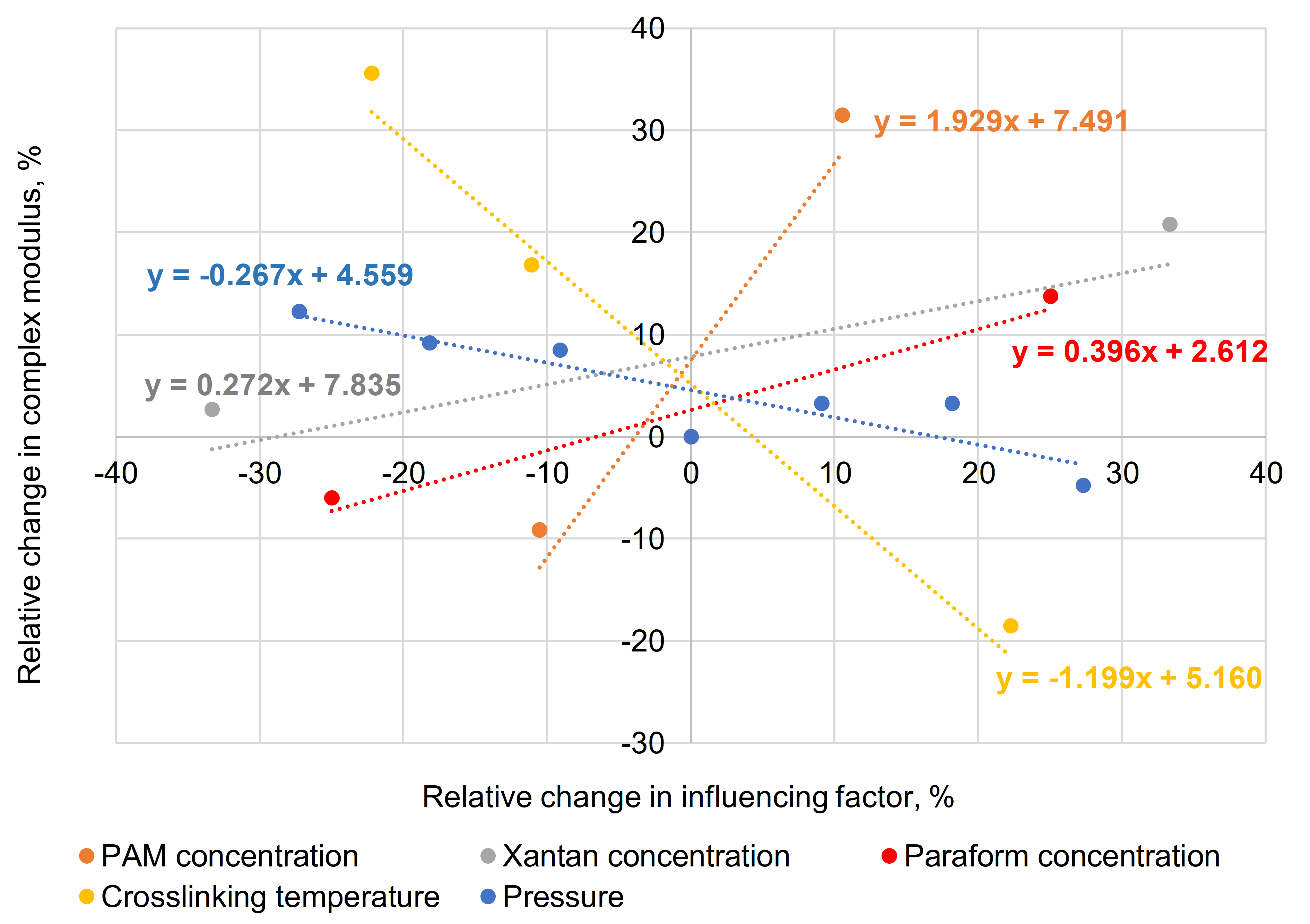

Supplement: Supplementary file 1 [file gels-11-00862-s001.zip › Figure_S7.png]

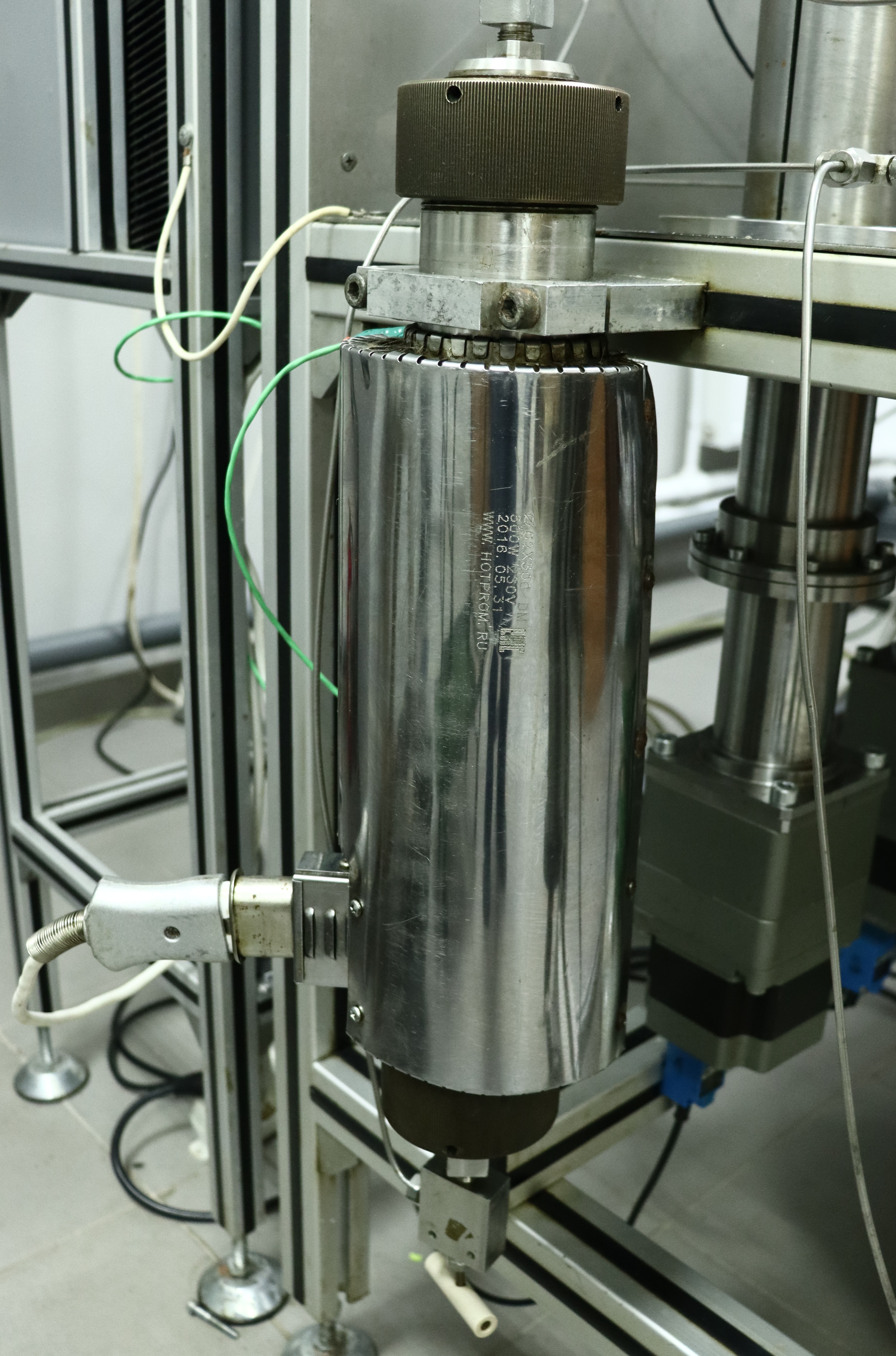

Supplement: Supplementary file 1 [file gels-11-00862-s001.zip › Figure_S8.png]

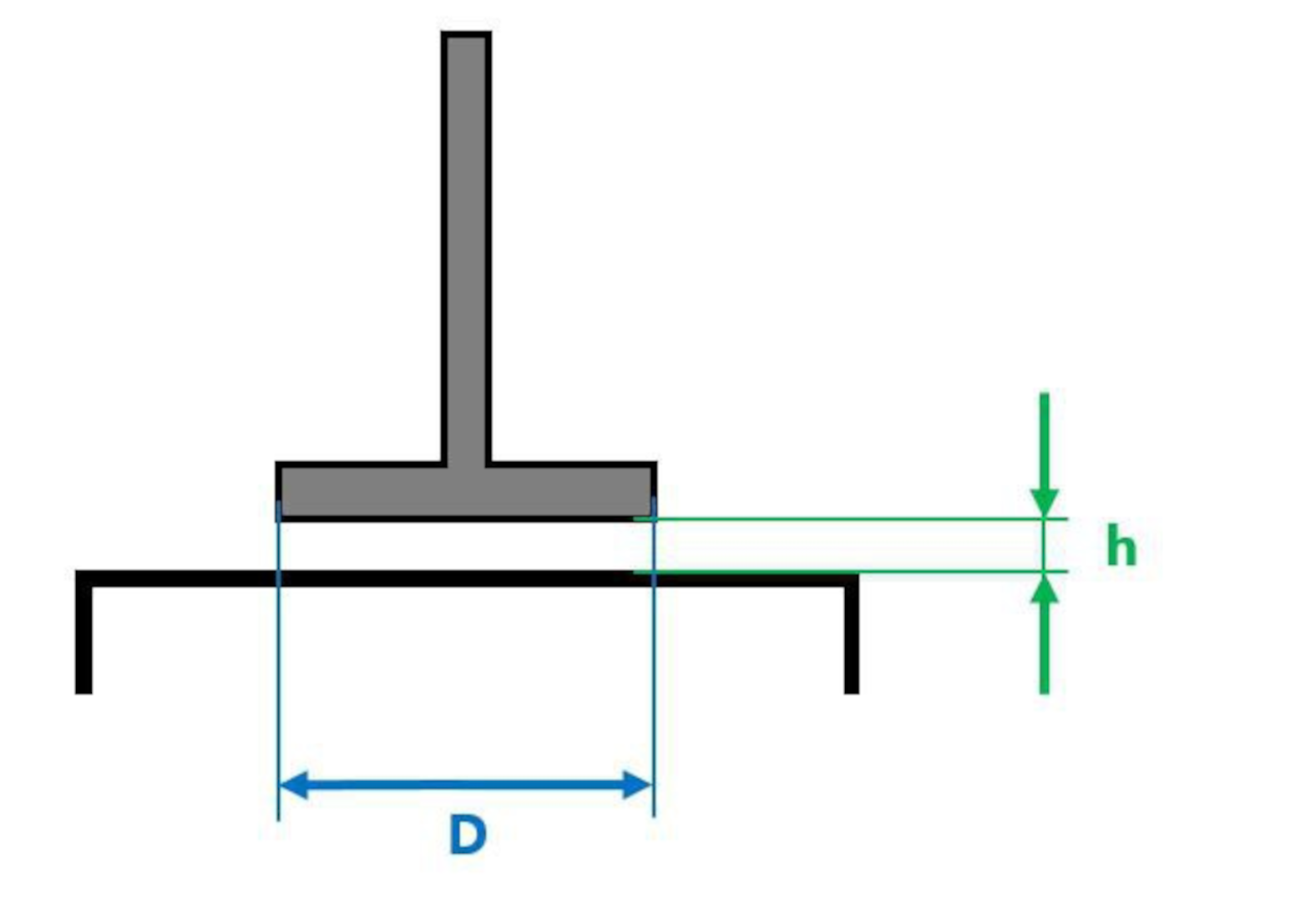

Supplement: Supplementary file 1 [file gels-11-00862-s001.zip › Figure_S9a.PNG]

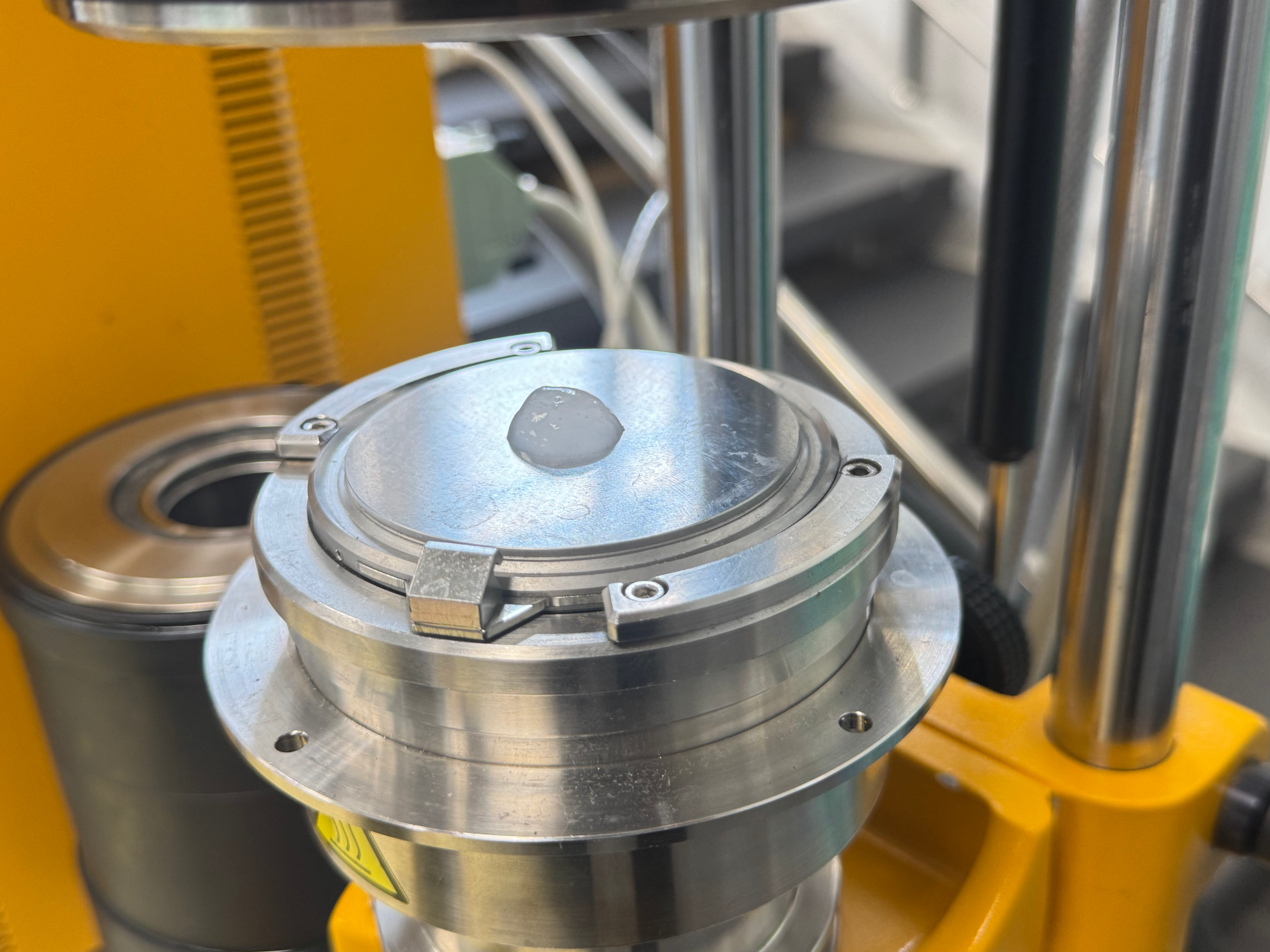

Supplement: Supplementary file 1 [file gels-11-00862-s001.zip › Figure_S9b.png]
